# Livestock enclosures in drylands of Sub-Saharan Africa are overlooked hotspots of N_2_O emissions

**DOI:** 10.1038/s41467-020-18359-y

**Published:** 2020-09-15

**Authors:** Klaus Butterbach-Bahl, Gretchen Gettel, Ralf Kiese, Kathrin Fuchs, Christian Werner, Jaber Rahimi, Matti Barthel, Lutz Merbold

**Affiliations:** 1 International Livestock Research Institute (ILRI), Mazingira Centre, PO Box 30709, Nairobi, 00100 Kenya; 2 Karlsruhe Institute of Technology, Institute of Meteorology & Climate Research (IMK-IFU), Kreuzeckbahnstr. 19, 82467 Garmisch-Partenkirchen, Germany; 3 IHE Delft Institute for Water Education, PO Box 3015, Westvest 7, 2611 AX Delft, The Netherlands; 4 ETH Zurich, Sustainable Agroecosystems, Department of Environmental Systems Science, Universitätsstrasse 2, 8092 Zürich, Switzerland

**Keywords:** Biogeochemistry, Climate sciences

## Abstract

Sub-Saharan Africa (SSA) is home to approximately ¼ of the global livestock population, which in the last 60 years has increased by factors of 2.5–4 times for cattle, goats and sheep. An important resource for pastoralists, most livestock live in semi-arid and arid environments, where they roam during the day and are kept in enclosures (or bomas) during the night. Manure, although rich in nitrogen, is rarely used, and therefore accumulates in bomas over time. Here we present in-situ measurements of N_2_O fluxes from 46 bomas in Kenya and show that even after 40 years following abandonment, fluxes are still ~one magnitude higher than those from adjacent savanna sites. Using maps of livestock distribution, we scaled our finding to SSA and found that abandoned bomas are significant hotspots for atmospheric N_2_O at the continental scale, contributing ~5% of the current estimate of total anthropogenic N_2_O emissions for all of Africa.

## Abstract (web summary)

Africa houses approximately one third of the global cattle, sheep and goat population. Here the authors show that manure accumulation in livestock enclosures can emit significant quantities of the greenhouse gas N_2_O for decades after abandonment, totaling 5% of continental anthropogenic N_2_O emissions.

## Introduction

Anthropogenic disturbance of the global nitrogen (N) cycle has led to significant increases of atmospheric N_2_O concentrations from pre-industrial levels of 270 ppbv to 331.1 ppbv in 2018^1^. This has been mainly attributed to the increasing use of synthetic fertilizers since the 1950’s to boost crop production^2^; however, N_2_O emissions associated with manure management is also one of the key drivers of atmospheric N_2_O increase since pre-industrial times^3,4^, with its current contribution estimated at 0.33 Tg N_2_O yr^−1^ or 3% of all anthropogenic N_2_O sources^5^. Africa contains 20%, 27%, and 32% of the global cattle, sheep, and goat populations^6^, with the majority of these populations roaming in semi-arid and arid environments (63%, 82%, and 70%, respectively). Forming the economic backbone of pastoralist communities, livestock numbers have increased dramatically in the last 60 years, e.g., cattle increased from ~120 Mio head in 1960 to >350 Mio in 2018^7^ (Supplementary Fig. 1). These livestock are typically allowed to roam during the day for grazing and are held in temporary enclosures (in Swahili boma; in Afrikaans kraal) during the night, which can be used and subsequently abandoned any time from 1 month up to ~10 years. How the increase in livestock numbers, lack of manure spreading, and boma use and abandonment may have affected continental N_2_O emissions remains uncertain.

Grazing by livestock leads to a significant concentration of N in excreta, especially at low to medium feed quality, as only 7–33% of the ingested N is metabolized and the remaining N is excreted as dung or urine^8,9^. This change in N cycling may be particularly important in the semi-arid and arid regions of SSA (as well as other parts of the world), where pastoralist communities practice livestock production in traditional ways (Supplementary Fig. 2). Pastoralist communities do not typically use manure for fertilizer, as they do not produce crops in large amounts, if at all. As a result, manure accumulates in bomas, which after years can be up to several meters in height. Bomas are periodically abandoned when communities decide to move owing to, e.g., changes in resource availability. Manure remains in place, forming islands of soil fertility high in C, N, and P, which ultimately increases spatial heterogeneity in savanna landscapes and supports increased plant biomass and forage quality for many decades^10,11^. Bomas remain visible in the landscape even after years of abandonment as grassy glades, which attract wildlife, particularly large herbivores, who in turn deposit dung and maintain bomas as nutrient hotspots for decades up to thousands of years^12,13^.

**Fig. 1:**
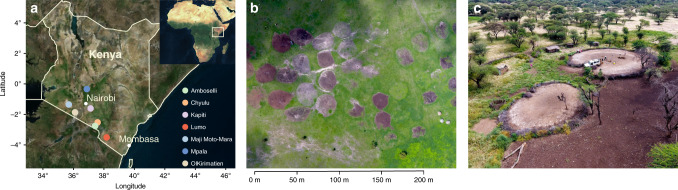
Location of study sites and aerial photos of bomas. **a** Position of measuring locations across Kenya; **b** aerial picture of bomas at Kapiti Research Station; and **c** measurement of N_2_O fluxes at boma sites at Ol Kirimatien Group Ranch in South-west Kenya.

Although a number of studies have focused on the importance of abandoned bomas as soil fertility hotspots and their role in landscape and ecosystem diversity^10–13^, the importance of bomas as possible landscape hotspots of soil N_2_O emissions remains unexplored. Other studies across various systems and climates have shown that N_2_O emissions are positively related to soil N availability^14^. To test the hypothesis that bomas are spatial hotspots of N_2_O emissions in semi-arid and arid regions of SSA at decadal time scales, we measured fluxes at abandoned bomas across an age gradient as well as in adjacent savanna sites in Kenya, East Africa (Fig. 1).

N_2_O fluxes were measured using the fast-box chamber method^15^ at seven different locations across Kenya at a total of forty-six boma sites, differing in age after abandonment (0.1–40 years) and at 22 adjacent savanna sites. In total, 257 flux measurements were conducted during the wet and dry seasons in 2018–2019 and were complemented by measurements of soil temperature and gravimetric water content. At all sites, we quantified the concentrations of soil C and N content at 0–10 cm depth, and at 0–1 m depth at a subset of sites (Kapiti Research Station, Mpala Research Centre, and Ol Kirimatien Group Ranch). Bomas that were abandoned from 0 to 15 years generally had no or little vegetation cover, likely a result of high nutrient concentrations. We therefore recorded vegetation presence and absence at each measuring site. We also observed an organic layer thickness of >0.3 m, indicating that the boma was used for consecutive years. In order to estimate measurement biases associated with day-time sampling, we investigated diurnal patterns of N_2_O emissions at a boma site at Kapiti Research Station. Furthermore, we explored short-term effects of rainfall on N_2_O fluxes by simulating a rain event by artificially adding water to a number of different bomas and adjacent savanna sites at the Mpala Research Centre.

Our results show that abandoned bomas remain active hotspots for N_2_O emissions for at least four decades, remaining about one order of magnitude higher than the savanna. Scaling this finding to SSA reveals that bomas are a neglected source for atmospheric N_2_O at the continental scale, with emissions more than doubling due to the increase in livestock in semi-arid and arid environments in the last 60 years. Based on our calculations, we estimate that bomas currently contribute about 5% of the current total anthropogenic N_2_O emissions for the continent of Africa.

## Results and discussion

### Comparing N_2_O fluxes from savanna and abandoned boma sites

In agreement with earlier studies^11,12^, we found higher C and N concentrations and stocks in the top 0–20 cm (in our study up to a magnitude higher) than in adjacent savanna soils, especially in bomas that were used for more than one year (Supplementary Table 1 and Supplementary Fig. 3). N_2_O fluxes at boma sites (*n* = 46) varied over five orders of magnitudes from 9.3–10,266 µg N_2_O-N m^−2^ h^−1^ (mean: 643 ± 255 µg N_2_O-N m^−2^ h^−1^, *n* = 187 flux measurements in total), whereas fluxes at savanna sites (*n* = 22) had lower variation and were on average two orders of magnitudes lower (mean: 5.5 ± 1.0 µg N_2_O-N m^−2^ h^−1^, range: –0.7 to 15.9 µg N_2_O-N m^−2^ h^−1^, *n* = 70 flux measurements). Boma sites without vegetation (*n* = 18) emitted significantly more N_2_O (981 ± 408 µg N_2_O-N m^−2^ h^−1^, *n* = 107) than older boma sites (*n* = 28), which had plant cover (119 ± 48 µg N_2_O-N m^−2^ h^−1^, *n* = 80). Bomas with an organic layer thickness > 0.3 m (*n* = 24) showed significantly higher N_2_O emissions (1125 ± 471 µg N_2_O-N m^−2^ h^−1^) than bomas with a shallow organic layer (*n* = 22) (119 ± 40 µg N_2_O-N m^−2^ h^−1^)^16^.

**Fig. 2:**
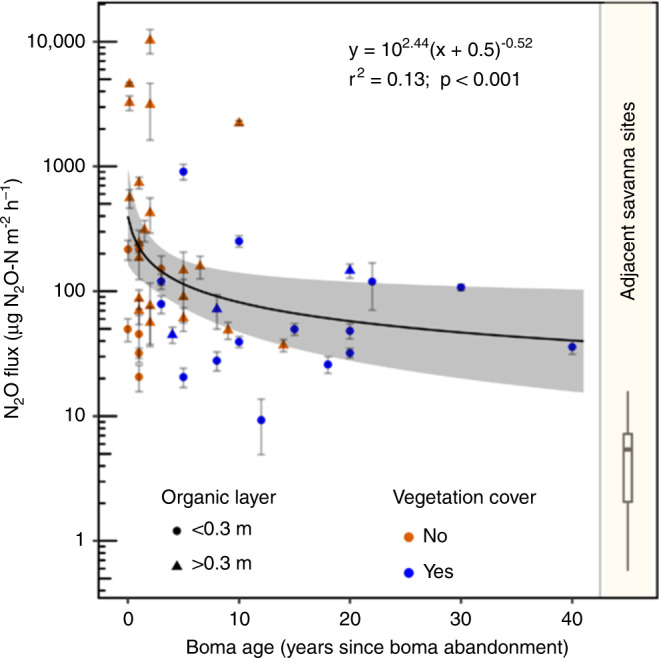
N_2_O fluxes from bomas (0.1–40 years old) and savanna sites. This figure shows data for bomas sampled in Kenya from 0.1 to 40 years in comparison with fluxes at adjacent savanna sites. Round symbols refer to boma sites with organic layer thickness of <0.3 m, whereas triangles indicate that the organic layer thickness is >0.3 m. Bomas without vegetation cover are marked by orange symbols, whereas blue symbols show bomas with vegetation cover. Details on the regression are given in Supplementary Table 2. The 95% confidence interval for the regression is highlighted in gray. Error bars indicate the standard error of the mean for each boma. Distribution of fluxes for adjacent savanna sites are summarized in a box plot (dark line depicts the median; box shows 25–75% percentiles, and whiskers depict range of measurements). Given their small magnitude, savanna sites were not considered as background in the boma flux calculations.

N_2_O fluxes declined exponentially with time since boma abandonment (Fig. 2), this also corresponded to a negative correlation of soil N content (%) with boma age (Supplementary Fig. 4), which was consistent with our hypothesis and previous studies^14^.

There was no relationship between N_2_O fluxes and soil moisture across the 46 boma sites in our data set. However, the simulated rainfall event enhanced average N_2_O fluxes by a factor of 3 for ~60 hours (from 20.0 ± 4.2 µg N_2_O-N m^−2^ h^−1^ to 56.1 ± 12.6 µg N_2_O-N m^−2^ h^−1^) (Supplementary Fig. 5). This contrasted with results observed for the neighboring savanna sites, which showed no enhancement of N_2_O fluxes with rainfall (before: 0.6 ± 0.5 µg N_2_O-N m^−2^ h^−1^, after simulated rainfall: 0.9 ± 0.5 µg N_2_O-N m^−2^ h^−1^). With respect to diurnal variation, measurements revealed that soil N_2_O fluxes were approximately two times higher in the afternoon than in the morning hours (Supplementary Fig. 6). We performed the measurements in this study in the morning hours and on days without rainfall, so we consider these measurements to be biased towards the low-end of potential N_2_O emission estimates.

### Bomas as sources of N_2_O at the continental scale

To assess whether our findings of elevated N_2_O fluxes from bomas are relevant at the scale of Africa and at decadal time-periods, we first calculated cumulative N_2_O emissions for a 40-year period following abandonment using the exponential regression curve of Fig. 2. We consider this to be a conservative estimate because we did not include the range of variation from diurnal measurements or pulse emissions of N_2_O following rainfall events. This calculation resulted in a cumulative mean emission rate of 25 g N_2_O-N per m^−2^ boma area over 40 years (95% confidence interval, CI: 13.3–48.1 g N_2_O-N m^−2^) and a mean annual rate of 0.62 g N_2_O-N m^−2^ (CI: 0.33–1.20 g N_2_O-N m^−2^ yr^−1^). In order to scale up these estimates to the continent, we multiplied the mean 40-year emission rate with the total abandoned boma areas in semi-arid and arid environments in sub-Saharan Africa (SSA), which we estimated for each year. The calculation of annual abandoned total boma area was based on information on animal numbers, area per livestock in bomas, average number of years of boma use, an average number of bomas used simultaneously per herder, and the fraction of bomas that were not extracted for manure (i.e., as fertilizer or for selling) (Supplementary Fig. 7). We developed a range estimate by simulating various scenarios for the combination of the different scaling parameters to explore overall uncertainty of our continental boma N_2_O emissions estimate by Monte Carlo simulation (see Methods, Supplementary Figs. 7 and 8).

**Fig. 3:**
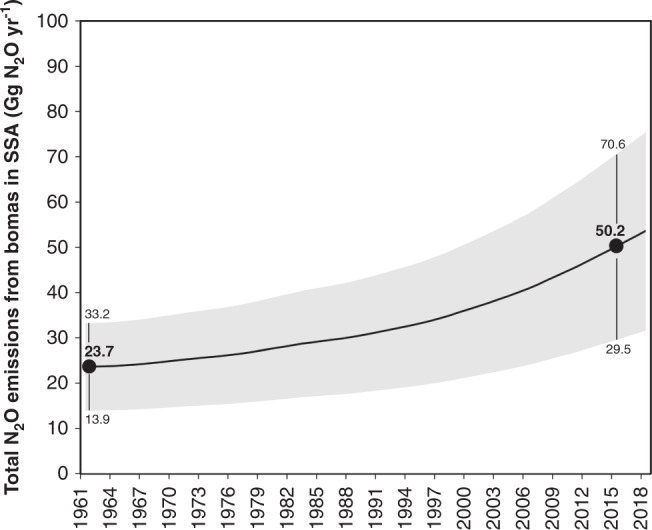
Change in the source strength of abandoned bomas from 1961 to 2018. The emission trend was based on changes in livestock numbers in semi-arid and arid SSA and the calculation scheme outlined in “Methods”. The bold line indicates the median value, while the gray shaded area represents the upper and lower quartiles.

**Fig. 4:**
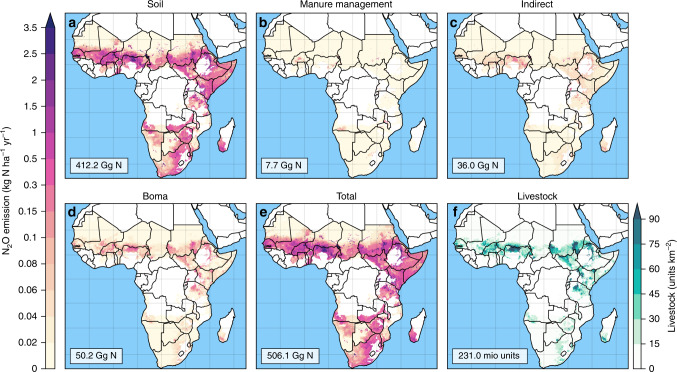
Sources of agricultural N_2_O fluxes and livestock units. Given are N_2_O fluxes for semi-arid and arid regions in SSA based on EDGAR5^5^ (**a**: soil; **b**: manure management; **c**: indirect emissions) and the total N_2_O emissions from bomas **d** from this study, and the resulting sum of total agricultural N_2_O emissions (=a+b+c+d) **e**. **f** depicts the livestock density for cattle, sheep, and goats in semi-arid and arid environments in Africa^6^. Livestock units^23^ summarize total number of cattle, sheep, and goats with $N = \left( {{\sum} {cattle} + \frac{{sheep \ast 0.1 + goats \ast 0.1}}{{0.7}}} \right)$.

According to our calculations, the median of total N_2_O emissions from bomas in semi-arid and arid climates of SSA is ~50.2 Gg N_2_O yr^−1^ (25–75th percentile: 29.5–70.6 Gg N_2_O yr^−1^, Fig. 3)^16^, with the highest N_2_O emissions in countries that have the highest numbers of livestock in pastoral systems, including Ethiopia, North- and South-Sudan, Kenya, Tanzania and Burkina Faso and Nigeria (Fig. 4). This is one magnitude higher than estimates of N_2_O emissions associated with manure management as documented in the EDGAR5 database for the same land area (7.7 Gg N_2_O yr^−1^) and comprises 5% of the current estimate of total anthropogenic N_2_O emissions for Africa at continental scale (EDGAR5 = 974 Gg N_2_O yr^−1^).

Our measurements of N_2_O emissions from bomas and resulting estimates for semi-arid and arid regions in SSA are relevant in the context of current uncertainties of anthropogenic sources and drivers of N_2_O increases in the atmosphere^17^, showing a systematic underestimation of N_2_O emissions from manure management at the scale of Africa. In 40 years following abandonment, an additional 1.34% (95% confidence interval, CI: 0.90–2.56%; Supplementary Note 1) of the accumulated manure N is directly emitted in form of N_2_O that is not considered by current IPCC methodology^18,19^. In addition, Africa’s population of livestock has increased exponentially in the last 60 years^7^ (Supplementary Fig. 1), and our results indicate that observed increases in atmospheric N_2_O concentration may not only be linked to the global increase in the use of fertilizers^20^, but also to numbers of livestock in semi-arid and arid regions and the manure accumulation in bomas (Fig. 4).

It was recently suggested that Africa contributed 20% to the observed increase in global N_2_O emissions between 2000 and 2005 and 2010 and 2015 (or ~0.32 Tg N yr^−1^ of the total 1.6 Tg N yr^−1^ increase)^17^. Our estimate of the increase in emissions from the abandoned bomas during this period is 0.006 Tg N yr^−1^ (or only 2% of this increase) and therefore cannot explain this difference. However, our measurements from abandoned bomas are biased low, and direct N_2_O emissions from active bomas and indirect N_2_O emissions from leached or volatilized N, which have not been investigated here, may have also contributed to the observed increase in N_2_O emissions from the African continent, and warrants further research.

Given the remoteness of settlements of pastoral communities, there is unlikely to be an economically viable solution for transporting and selling manure. However, simple management practices such as the use of mobile bomas, which can be changed at monthly intervals^21^, annual spreading or the distribution of manure upon abandonment on the adjacent rangeland, would not only significantly decrease N_2_O emissions, but could also work to restore rangeland productivity as nutrients are redistributed in the landscape, thereby creating larger areas with improved, nutrient-rich forage^22^.

## Conclusion

Our results show that N_2_O emissions from bomas have not been adequately considered, and therefore we recommend that current methodology for estimating N_2_O emissions from livestock held by pastoralists in semi-arid and arid landscapes in SSA and elsewhere should be refined to include abandoned bomas. Moreover, further research in understanding indirect emissions due to N losses from bomas along hydrological (leaching, surface run-off) and atmospheric (mainly NH_3_ volatilization) pathways is needed to develop a full picture of the importance of abandoned bomas as a source for atmospheric N_2_O. To decrease the environmental N footprint of bomas, improved but simple manure management measures should be considered.

## Methods

### Field N_2_O flux measurements

At seven different semi-arid and arid savanna regions in Kenya (Fig. 1, Supplementary Fig. 9), N_2_O measurements from soils at 46 boma sites and 22 adjacent reference sites (undisturbed savanna) were carried out. At each boma and control site, 3–7 plots were chosen randomly for flux measurements. Local members of the pastoral communities, including herders and/or community elders were interviewed for information on the time since abandonment of each boma.

N_2_O fluxes from bomas were measured using the fast-box chamber method^15^, deploying an ultra-portable greenhouse gas analyzer of ABB-Los Gatos Research Inc. (Modell 909–0041). A gas-tight, vented chamber (0.3 × 0.2 × 0.15 m) was pressed against the ground on foam frames for 4–7 min, during which time sample air was pumped from the headspace of the chamber to the analyzer and returned to the chamber thereafter. In this way, changes in headspace N_2_O concentrations were continuously measured over the sample period, with a running average of every 5 s. Linear regression over the sample period was used to calculate fluxes. The detection limit for N_2_O fluxes was <1 µg N_2_O-N m^−2^ h^−1^.

### Soil and environmental parameters

Environmental variables including gravimetric and volumetric soil water content, soil and air temperatures, organic layer thickness, and aboveground plant biomass (if present) were measured in all plots. At selected boma sites, we also measured total N and C stocks for 0–1 m depth (see Supplementary Table 1 and Supplementary Fig. 9). We noted crusts only on bomas that were abandoned for <5 years. As nutrient concentrations diminish with time, vegetation starts to grow on abandoned bomas. Furthermore, owing to rainfall and trampling of wild animals and sometimes of livestock, crusts are fragile, fragmented and <5 mm in thickness.

### Identifying drivers of N_2_O fluxes

For regression analyses on controlling parameters of N_2_O fluxes (Supplementary Tables 2–4; Supplementary Notes 2 and 3), we calculated an average flux per boma (or control) site for our analyses. We used Welch’s *t* test to compare N_2_O fluxes from boma and adjacent savanna plots, as well as for comparing N_2_O fluxes from vegetated plots with those from bare soil plots and from plots of high versus low organic layer depth (>0.3 m versus <0.3 m). A depth of 0.3 m was chosen as a threshold value to distinguish between bomas that were used for 1–2 years and those that were used consecutively for >2 years, as manure accumulates at a rate of 0.1 to 0.15 m yr^−1^.

To determine the effect of potential driver variables on N_2_O fluxes (log10), we explored a multiple regression model for gravimetric soil water content (including a quadratic term to allow a decrease at high soil water content) as well as soil temperature, organic layer N, organic layer C, vegetation cover, time since abandonment, and organic layer depth (Supplementary Table 3 and 4, multiple *r*^2^ = 0.41, adjusted *r*^2^ = 0.26). Only soil temperature significantly affected N_2_O fluxes in the multiple linear regression model. In addition, a mixed modeling approach was used to assess whether random effects (e.g., on site) improved the model. This did not improve the model performance, indicated by a higher AIC (138 versus 91).

To estimate overall N_2_O emissions of bomas over time, we built a simple regression model relating boma age (since abandonment) to average boma N_2_O fluxes on a log-log scale (Supplementary Table 2, displayed in Fig. 2). Since the variability explained by this model is moderate (*r*^2^ = 0.13), there are relatively wide confidence intervals (Fig. 2), but these reflect the full variability of our measured N_2_O fluxes. From the model fit, we calculated the cumulative N_2_O emission on a per-area basis over 40 years, i.e., including oldest abandoned bomas investigated.

### Upscaling N_2_O fluxes from bomas to SSA

The estimated N_2_O flux over the 40 years period served as an input for upscaling measurements, thereby taking into account uncertainty of estimated N_2_O flux as well as the uncertainty in further parameters used for spatial upscaling (Supplementary Fig. 7 and below).

Gridded estimates of livestock numbers were taken from Gilbert et al.^6^ (Fig. 4f) and restricted to semi-arid and arid environments of SSA (Supplementary Fig. 10). The calculation of total livestock units for pastoral systems (i.e., sum of cattle+goat+sheep) was based on livestock conversion factors as reported by Houerou and Hoste^23^, which are for cattle in herd 0.7 and for sheep and goat 0.1. Information on the boma area per livestock unit was obtained from Okello et al.^24^. Based on interviews with herders and local experts it became evident that several bomas are used at the same time as herders move their livestock across grazing areas in response to the availability of forage and water and that at least two bomas are simultaneously in use on average—one close to a semi-permanent homestead and one within seasonal grazing areas. The number of years that a boma is used varies considerably across sites, between 1 month to up to several years in this study and confirmed by several others^12,25^, with 3.7 years being the central value. Information on manure extraction from bomas was not available. Based on interviews with herders we assumed for 90% of bomas manure is either not extracted or irregularly or only partially extracted. Manure use increases in regions with mixed farming, i.e., simultaneous crop and livestock production and short (<2–5 km) transport distances, and with increasing humidity^26^, but is hardly existing in remote regions with livestock production only, which are in the focus of our study.

Total N_2_O emissions from bomas in semi-arid and arid environments of SSA were calculated as follows:

Step 1: Calculation of total livestock numbers (TLN) by combing population data for cattle, goat and sheep and converting to a single livestock unit, based on conversions for semi-arid and arid environments^23^.

$${\boldsymbol{TLN = }}{\sum} {{\boldsymbol{cattle}}} + \frac{{({\boldsymbol{sheep}} \times 0.1 + {\boldsymbol{goat}} \times 0.1)}}{{0.7}}$$

Cattle, sheep, goat: total number of cattle, sheep, goat

Step 2: Calculating boma use intensity (BUI)

***BUI*** = BAL * NB * FMB/YB

with

*BAL:* boma area per livestock, the work of Okello et al.^24^ indicates that the space for one cattle in a boma is ~4–16 m^2^, central value: 10

*NB:* number of bomas in use at the same time, interviews and expert knowledge, central value = 2.5

*FMB:* Fraction of bomas without use of manure, interviews and expert knowledge, central value = 0.9

*YB:* years of boma use, interviews and expert knowledge, central value = 3.7

Note: in 2015 the total area of newly abandoned bomas in semi-arid and arid SSA was estimated at 1792 km^2^. The total area of semi-arid and arid SSA equals 15.16 Mio km^2^. Thus, the area of newly abandoned bomas equals 0.11‰ of the total area of semi-arid and arid SSA.

Step 3: Calculating N_2_O emission intensity (N_2_O_int)

*N*_*2*_*O_int = N*_*2*_*O x N*_*2*_O_years * 4/28

*N*_*2*_*O:* mean average annual N_2_O flux from bomas, this study

N_2_O_years = observed minimum number of years with N_2_O fluxes significantly greater than at adjacent savanna sites = 40 years

44/28 = conversion of N_2_O-N to N_2_O

Step 4: Calculating total N_2_O emissions from bomas in semi-arid and arid environments

$${\sum} {N_2} O\,from\,bomas\left( {Gg\,N_2O} \right) = \frac{{TLNx\,BUI\,x\,N_2O\_int}}{{1000000000\left[ { = conversion\,g\,to\,Gg} \right]}}$$

Or (summarizing steps 1–3)

$$\begin{array}{l}{\sum} {N_2} O\,from\,bomas\left( {Gg\,N_2O\,over\,40\,years} \right) = \\ \frac{{TLNx\,BAL\left( {m^{ - 2}} \right)x\,NB\,x\,FMB\,x\,N_2O\left( {\mu g\,N_2O - N\,m^{ - 2}yr^{ - 1}} \right)x\,40[ = years\,of\,N_2O\,flux\,activity\,after\,abandonment]}}{{YB\left( {years} \right)^\ast 1000000000\left[ { = conversion\,to\,Gg} \right]x\left( {\frac{{28}}{{44}}} \right)[ = conversionN_2O - N\,toN_2O]}}\end{array}$$

Supplementary Fig. 7 shows histograms, probability distribution functions, and cumulative distribution functions of the different variables used for assessing the uncertainty of the upscaling procedure. To provide statistics such as median, 25 and 75% percentile values, etc. for upscaled N_2_O emissions we used the Latin hypercube sampling (LHS) method. The concept behind LHS is to divide the cumulative curve of each variable into *n* equally probable intervals and take a random sample at each probable interval for each variable. The *n* values obtained for each of the components were then paired with each other to recalculate the equation n times for assessing the uncertainty in a prediction equation.

## Supplementary information

Supplementary Information

Peer Review File

Supplementary Data

## Data Availability

The datasets generated during the current study are available in the figshare repository 10.6084/m9.figshare.12793868. Raw data files and graphic files are available from the corresponding author upon reasonable request.
