# Supplementary material for: Livestock enclosures in drylands of Sub-Saharan Africa are overlooked hotspots of N2O emissions
Source: Nat Commun. 2020 Sep 15;11:4644. doi: 10.1038/s41467-020-18359-y (PMC7493980; doi:10.1038/s41467-020-18359-y)
Supplement: Supplementary file 1 — Supplementary Information [file 41467_2020_18359_MOESM1_ESM.pdf]

Supplementary Information for "Livestock enclosures in drylands of Sub-Saharan Africa are overlooked hotspots of N_2O emissions by Butterbach-Bahl et al."

Supplementary Figures

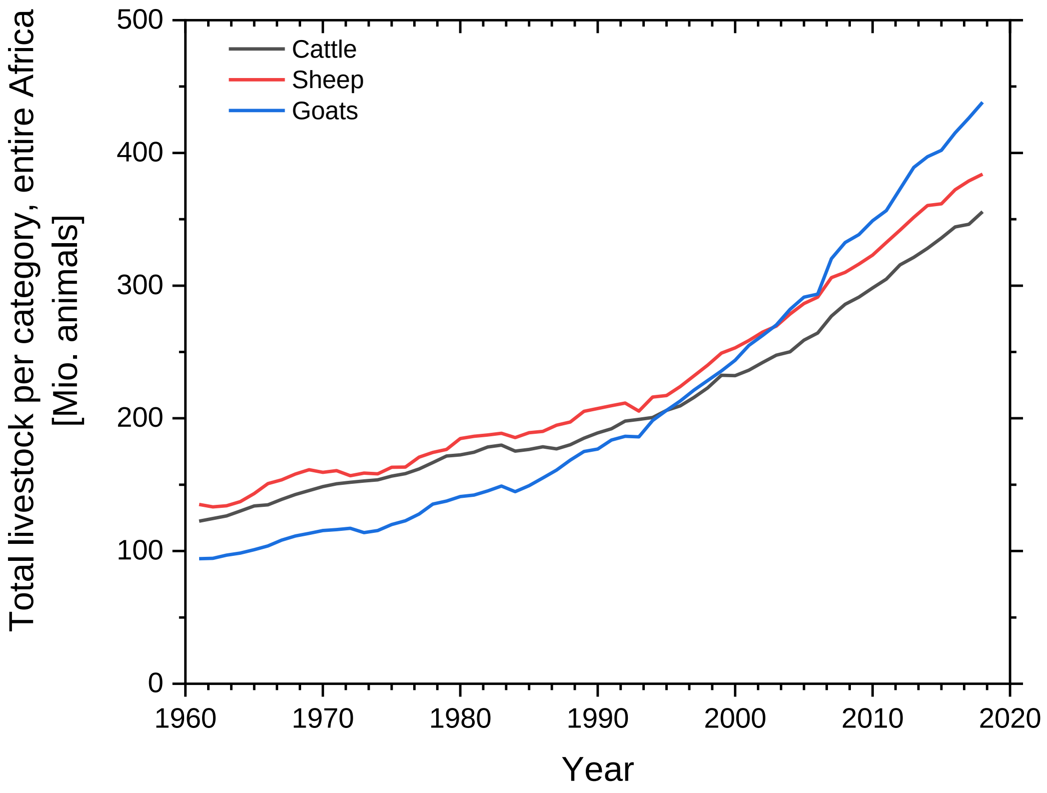

Supplementary Figure 1. Changes in total livestock numbers on the African continent from 1961 – 2018. Data from FAO[1].

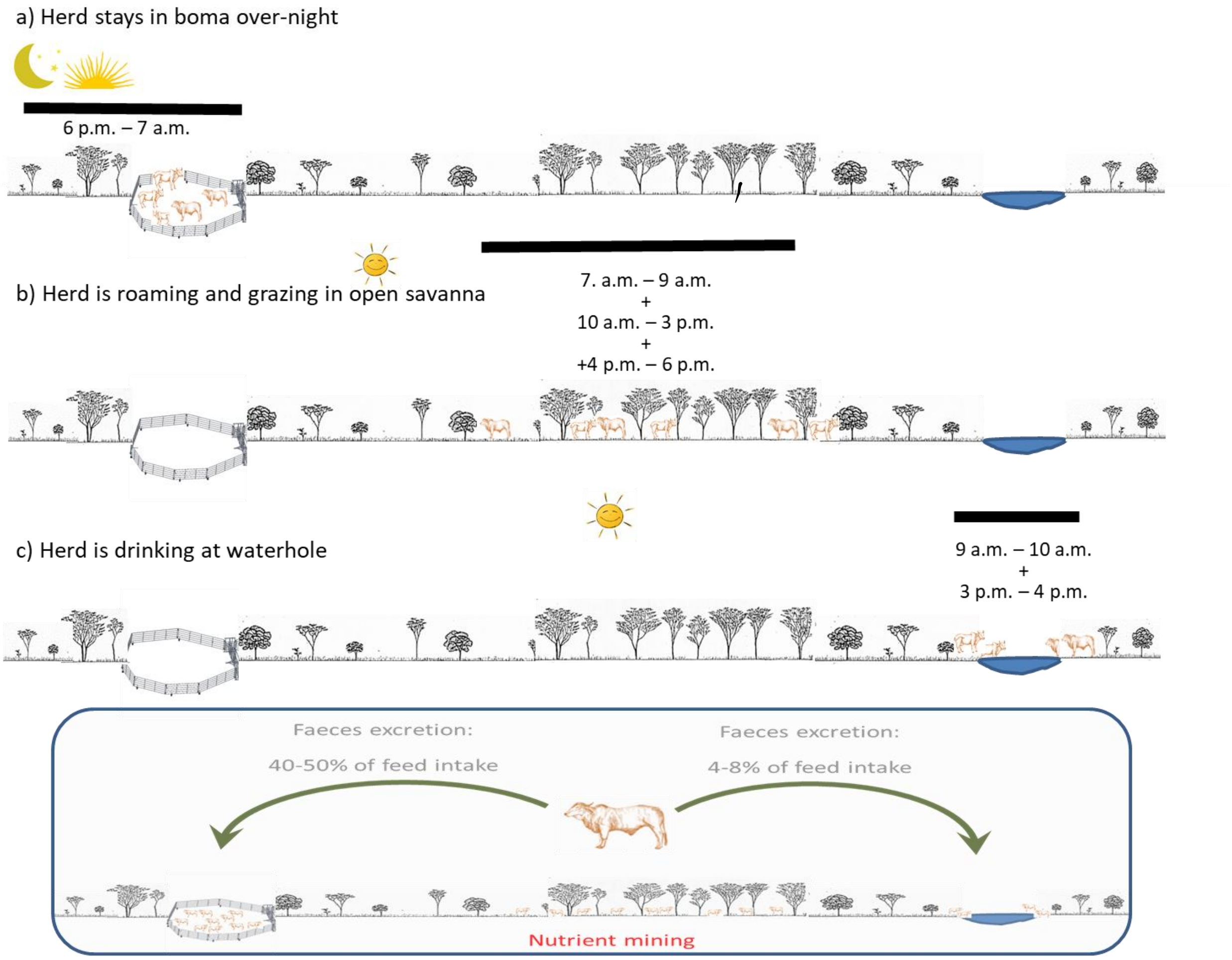

Supplementary Figure 2. Simplified scheme of herd management by pastoralist. The figure outlines general herd management activities in semi-arid and arid savanna landscapes and resulting nutrient translocation from savannas to bomas (and watering sites).

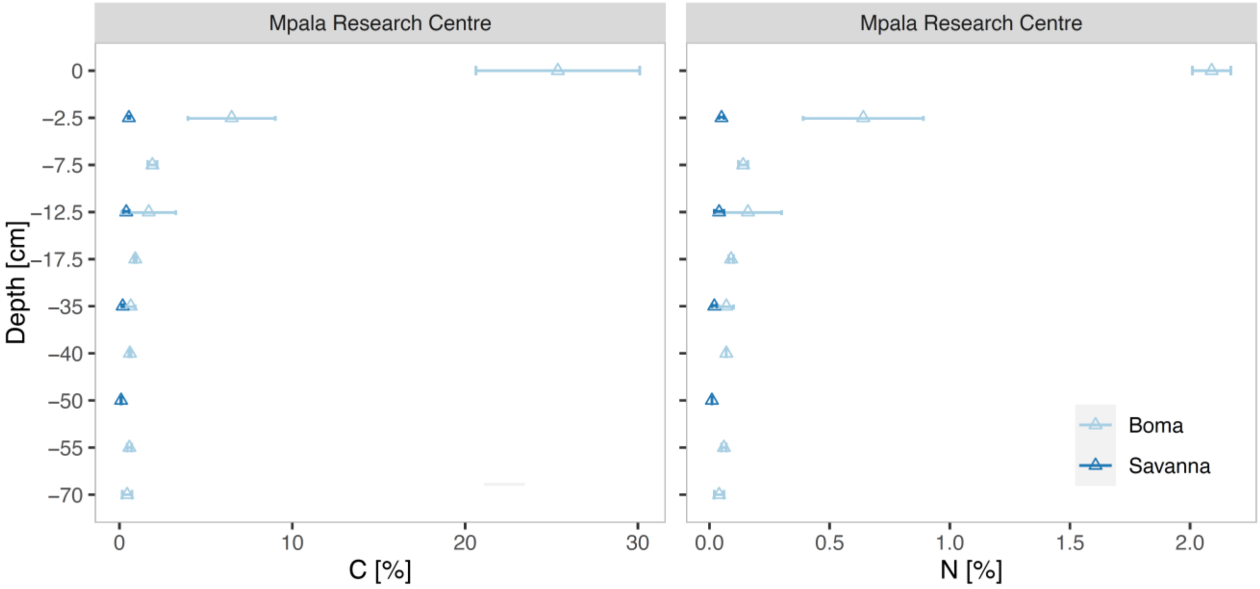

Supplementary Figure 3. Depth profiles of soil carbon (C) and nitrogen (N) concentrations. Given are mean values (± SD) of soil C and N concentrations as measured at five *boma* and five adjacent savanna sites in Laikipia county (Mpala Research Centre), Kenya. For this analysis *boma* chronosequence data were pooled (see Table S1).

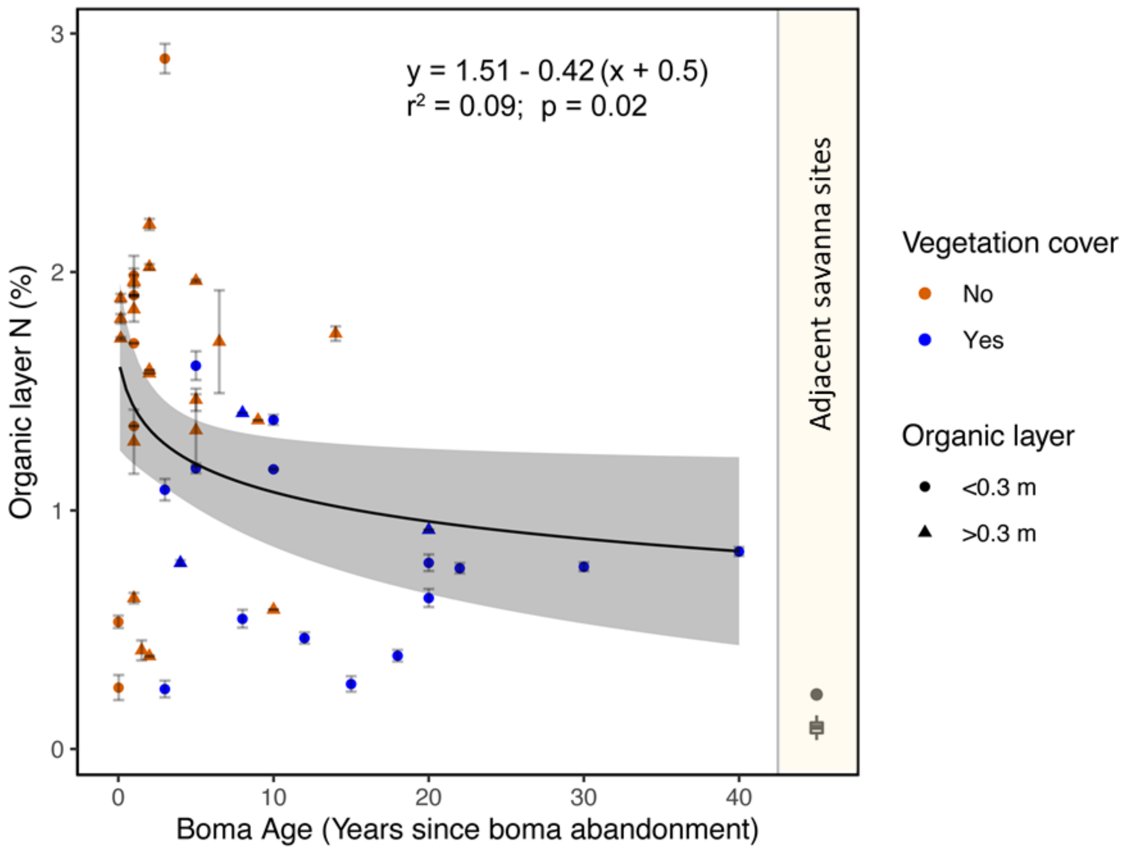

Supplementary Figure 4. Topsoil N concentration at bomas (0.1-40 years old) and savanna sites. Round symbols refer to sites with an organic layer thickness of <0.3m, while triangles indicate that the organic layer thickness is >0.3 m. *Bomas* without vegetation cover are marked by orange symbols, while blue symbols show *bomas* with vegetation cover. The 95% confidence interval for the regression is highlighted in grey. Error bars indicate the standard error of the mean for each *boma*. Details on the regression are given in Table S1. Distribution of topsoil N concentrations at adjacent savanna sites are summarized in a box plot (dark line depicts the median; box shows 25% - 75% percentiles, and whiskers representing 1.5 times the interquartile range).

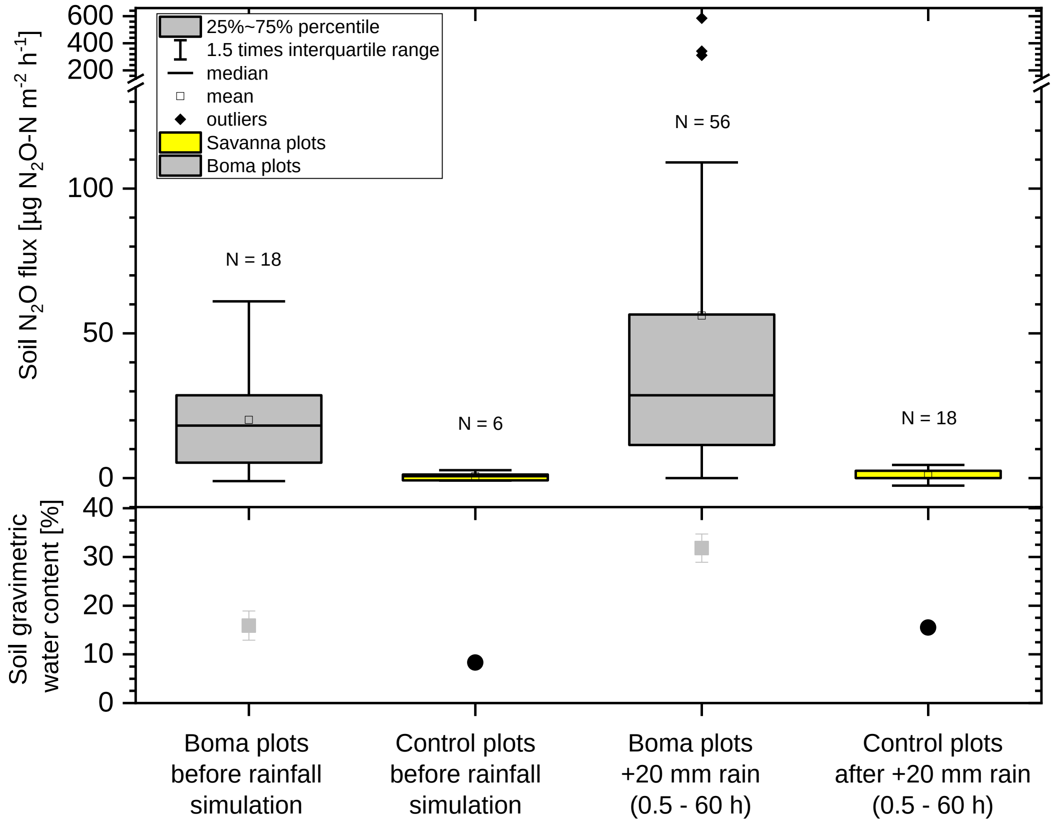

Supplementary Figure 5. Effect of rainfall on N_2O fluxes at boma and savanna sites. Shown are changes of soil mean N_2O fluxes at three *boma* sites in Laikipia county at Mpala Research Centre (age since abandonment 5, 8 and 12 years, 3 replicated plots each) and adjacent savanna sites (N=3, each with 3 replicated plots) following a simulated rainfall of 20 mm. We show N_2O emissions before and after rainfall, with N representing the number of flux measurements. N_2O fluxes after simulated rainfall (sprinkling of plots with water equivalent to 20 mm rainfall) were observed over a 60h period.

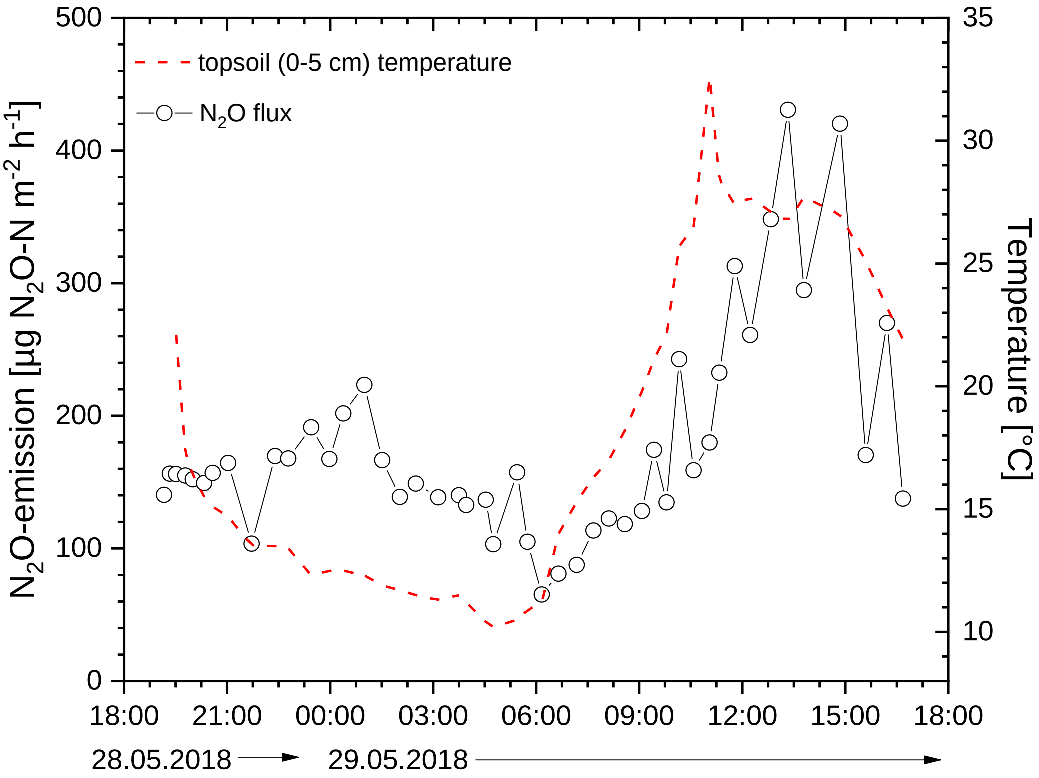

Supplementary Figure 6. Diurnal variability of N_2O fluxes and topsoil (0-5 cm) temperature at a boma site. Shown are measurements of N_2O fluxes over a 24h period as measured at a *boma* site at Kapiti Research station, Machakos county, Kenya. N_2O fluxes were measured applying the fast-box chamber method. Here a large chamber (1.5m x 1.5m x 0.3m) was closed for approx. 3 min and re-opened thereafter. Measurements were replicated every 30min for 24 hours. Linear increases in headspace N_2O concentration as measured by an ultra-portable greenhouse gas analyzer (ABB-Los Gatos Research, Modell 909-0041) were used to measure N_2O concentrations.

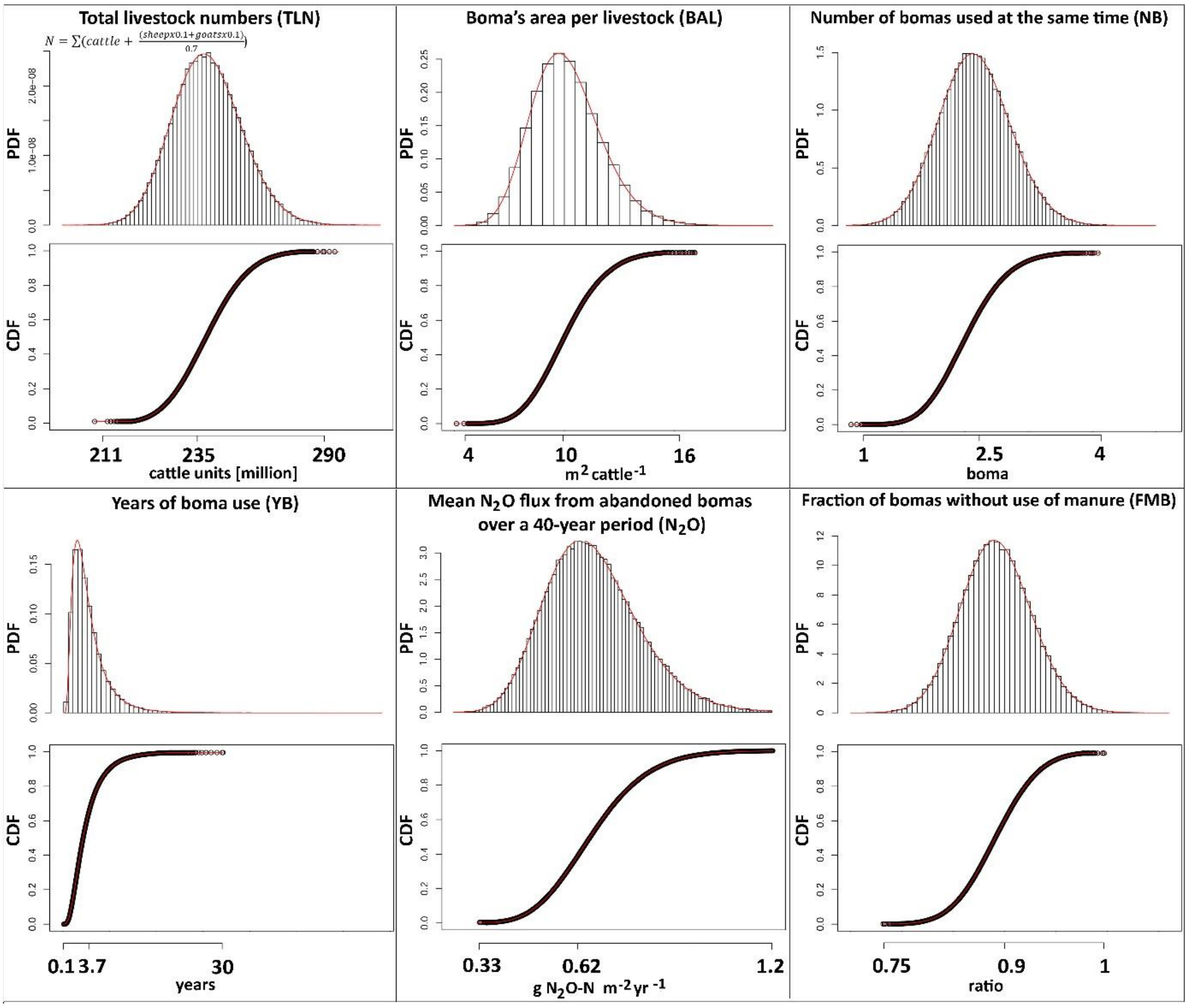

Supplementary Figure 7. Probability distribution functions for parameters used to upscale N_2O fluxes. Shown is the estimated uncertainty of parameters (Probability Distribution Functions (PDF), and Cumulative Distribution Functions (CDF)) used to upscale N_2O fluxes from boma sites to semi-arid and arid regions in Sub-Sharan Africa (SSA). Mean values and standard errors of N_2O emissions from bomas at the scale of SSA were calculated by considering the frequency distribution of parameters using a Monte Carlo approach.

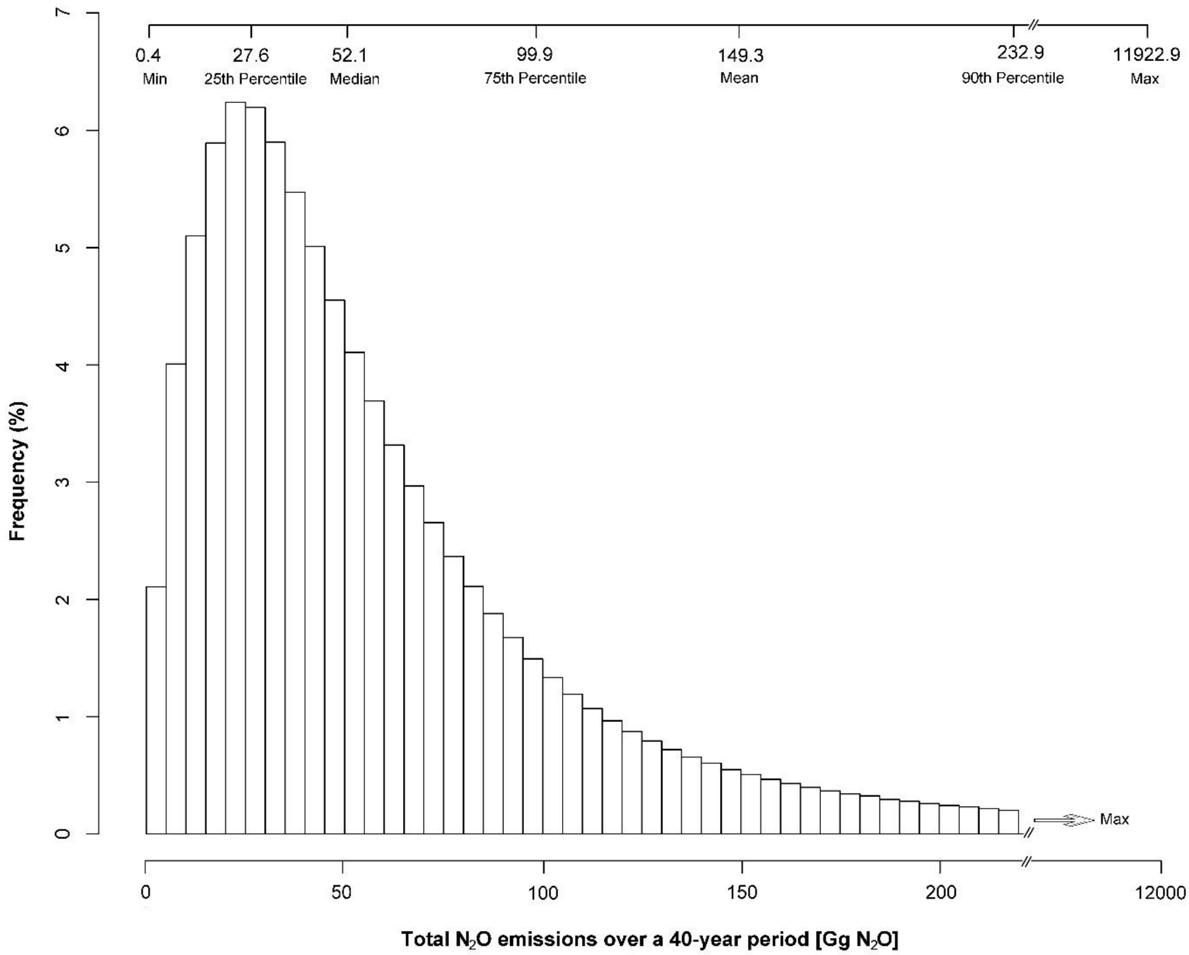

Supplementary Figure 8. Frequency distribution of upscaled boma N_2O emissions. N_2O emission strength at the scale of semi-arid and arid regions of SSA was calculated on basis of livestock numbers for 2010 (cattle, sheep and goats)[3], mean N_2O emissions over a 40 years period (Figure 2), boma usage time, and area per livestock in a *boma* (see Supplementary Figure 8).

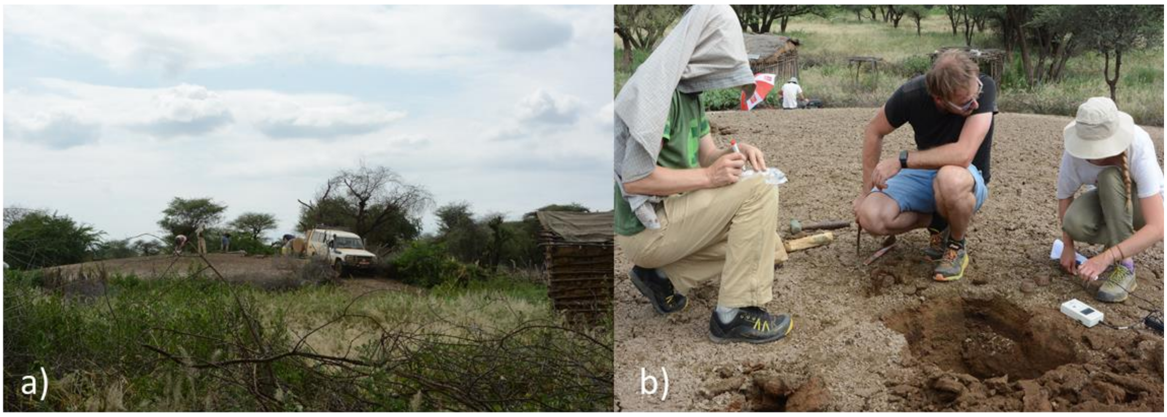

Supplementary Figure 9. N_2O flux measurements at an abandoned boma site. Pictures were taken at OlKirimatien Group Ranch in South-West Kenya (see also Figure 1c in the main text). a) A view of a boma site with 6 m manure accumulation. This boma was used for 8 – 10 years for sheep and goats. b) Manure sampling for further laboratory analysis.

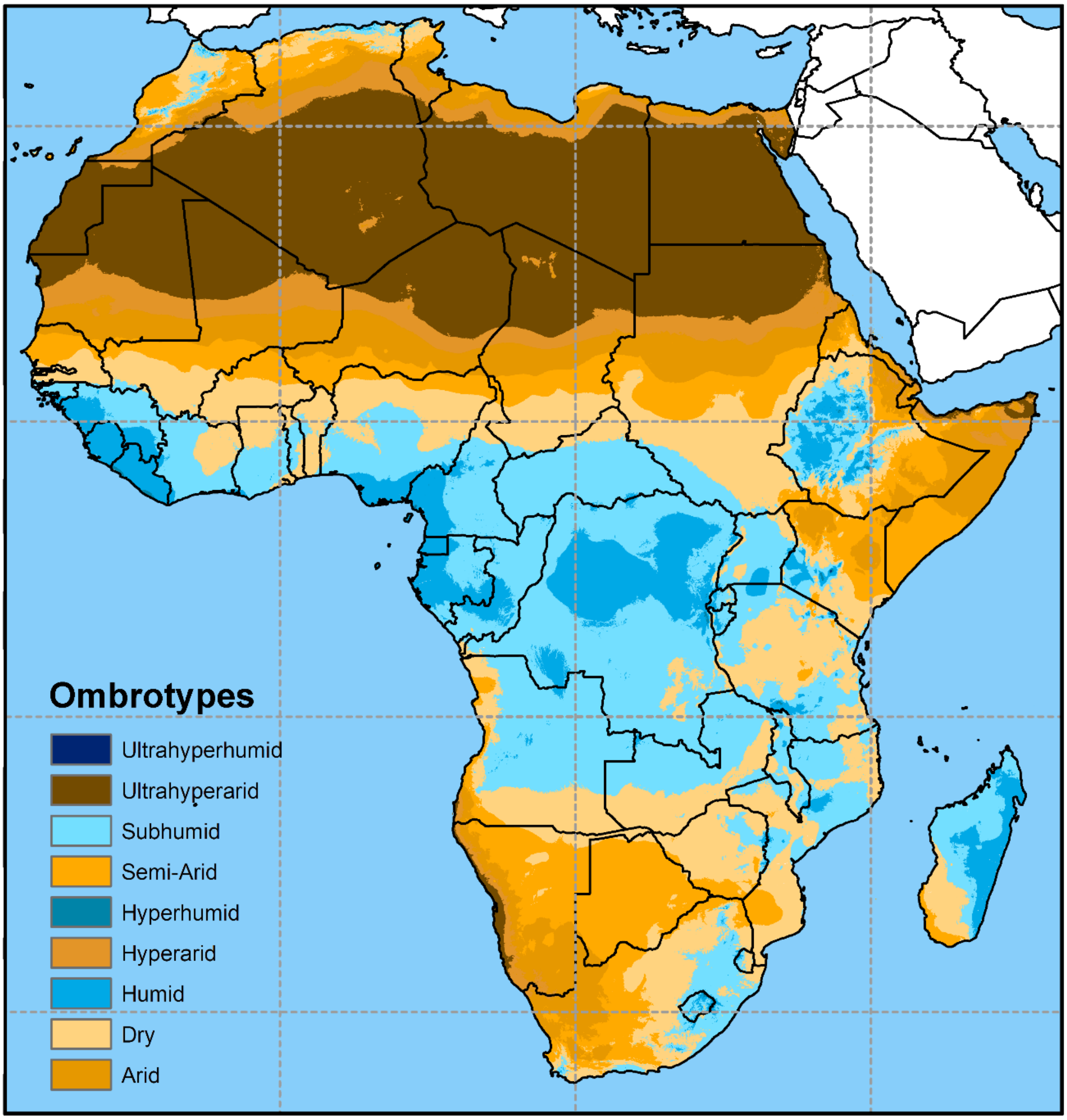

Supplementary Figure 10. Regional distribution of climate zones in Africa[2]. The analysis was restricted to hyper-arid, arid, semi-arid, arid and dry zones.

Supplementary Tables

Supplementary Table 1. Soil carbon (C) and nitrogen (N) stocks in bomas and savanna. Mean (± SD) of soil C and N stocks down to 1 m soil depth were calculated from measured concentrations and bulk density values at a chronosequence of bomas and adjacent savanna sites in Laikipia county (Mpala Research Centre), Kenya.

**Boma Age**	**C stock**	**N stock**
	Mg C ha^{-1}	Mg N ha^{-1}
**10**	154.7 ± 23.9	15.1 ± 2.3
**15**	63.9 ± 9.6	6.6 ± 0.8
**20**	104.4 ± 24.7	10.5 ± 2.1
**30**	144.3 ± 11.5	14.8 ± 1.0
**40**	175.6 ± 27.3	17.5 ± 2.4
**Savanna Reference**	26.6 ± 4.7	2.8 ± 0.5

Supplementary Table 2. Regression equations. Given are the relationships between time since abandonment (Boma age) and N_2O fluxes, between time since abandonment (Boma age) and organic layer N, and between organic layer N and N_2O fluxes. All effects were highly significant ($p < 0.001$), as indicated by stars ***; The p-value refers to the overall model fit.

y	x	Linear model	b0 (± SE)	b1 (± SE)	r^2_{adj}	p-value	Back-transformation	degrees of freedom
N_2O flux [µg N m^{-2} h^{-1}]	Boma Age [yr^{-1}]	$\log_{10}(y)$ = b0 + b1 $\log_{10}(x + 0.5)$	2.44*** (±0.15)	-0.52*** (±0.18)	0.13	0.007	$y = 10^{b0}(x + 0.5)^{b1}$	44
Soil N [%N]	Boma Age [yr^{-1}]	y = b0 + b1 $\log_{10}(x + 0.5)$	1.51*** (±0.14)	-0.42*** (±0.18)	0.09	0.02	y = b0 + b1 $\log_{10}(x + 0.5)$	44
N_2O flux [µg N m^{-2} h^{-1}]	Soil N [%N]	$\log_{10}(y + 1)$ = b0 + b1 x	0.99 *** (±0.13)	0.77*** (±0.11)	0.40	$4.7\ 10^{-9}$	$y = 10^{b0}\ 10^{b1\,x}$-1	66 (includes bomas + control)

Note: For modeling the relationship of organic layer N and of boma age with N_2O fluxes, the N_2O flux was log-transformed (log10). For modeling the effect of boma age on organic layer N, additionally the explanatory variable boma age was log-transformed.

Supplementary Table 3. Multiple regression model to identify drivers of N_2O emissions from bomas

log10(N_2O-Flux [µg N $m^{-2}h^{-1}$]) ~ Gravimetric soil moisture [g g^{-1}] + I(Gravimetric soil moisture [g g^{-1}])^2 + Soil temperature [°C] + Organic layer N [%] + Organic layer C [%] + Vegetation present [-] + Age class [-] + Organic layer depth [-]

	Unit	Estimate	SE	t-value	Pr(>|t|)	
Intercept	[-]	0.5561	0.6845	0.81	0.42	
Gravimetric soil moisture	[g g^{-1}]	0.0154	0.0099	1.56	0.13	
Gravimetric soil moisture^2	[g g^{-1}]^2	-0.0001	0.0001	-1.71	0.10	
Soil temperature	[°C]	0.0473	0.0207	2.28	0.03	*
Organic layer N	[%]	0.0373	0.2463	0.15	0.88	
Organic layer C	[%]	-0.0080	0.0160	-0.50	0.62	
Vegetation present	[-]	-0.2440	0.2877	-0.85	0.40	
Age class (10,20]	[-]	-0.4400	0.2809	-1.57	0.13	
Age class (20,40]	[-]	0.0082	0.3898	0.02	0.98	
Organic layer depth > 0.3 m	[-]	0.2457	0.2441	1.01	0.32	

Residual standard error: 0.5819 on 36 degrees of freedom

Multiple R-squared: 0.408, Adjusted R-squared: 0.26

F-statistic: 2.757 on 9 and 36 DF

p-value: 0.0146

Residual standard error: 0.4008 on 26 degrees of freedom

Reference Age class is up to ten (0-10] years

Vegetation present, Age class and Organic layer depth > 0.3 m are categorical variables

Supplementary Table 4 Cross correlation of continuous variables used in Supplementary Table 3

	Gravimetric soil moisture [g g^{-1}]	Soil temperature [°C]	Organic layer N [%]	Organic layer C [%]
Gravimetric soil moisture [g g^{-1}]	1.000			
Soil temperature [°C]	-0.041	1.000		
Organic layer N [%]	0.540	0.072	1.000	
Organic layer C [%]	0.408	0.100	0.793	1.000

Supplementary Notes

Supplementary Note 1. Calculation of the EF_{N2O} for abandoned bomas. Here we outline the assumptions and calculations on which the EF_{N2O} is based:

a) Fifty percent of the ingested feed is excreted in bomas as they are used approximately 12 hr day^{-1} (Figure 2);

b) Cattle dry matter intake is 2% of its body weight, which is an estimate for livestock feeding on dry pasture[3];

c) The average weight of a livestock (cattle = 1, sheep and goats = 0.1) unit is 300 kg. This was based on the mean between the IPCC estimate, which assumes mean live weights for dairy and other cattle in Africa to be 260 and 236 kg respectively (IPCC 2019, Chapter 11, Table 10.5)[4], and more refined estimates of Oklahoma State University[5] who found that Massai livestock were between 360 kg (cows) and 400 kg (bulls).

d) Livestock feed comprises mainly tropical grasses, which have a leaf dry matter nitrogen content ranging 0.6 - 1.7% (central value: 1.15%)[6].

e) Based on these numbers we calculated total N intake per cattle as follows:

   N_{intake} = 300,000 g (body weight) * 0.02 (%DM intake day^{-1}) * 0.0115 (%N in dry matter) = 69.0 g N day^{-1} (range 36 – 102 g N day^{-1});

   We estimate excretion using the assumption that N intake equals N excretion. This is based on the IPCC (2019) data for mature males held in low productivity systems and fed on pastures[4]. Although mature females have a N retention of 8%, we ignored this for the sake of this calculation. Thus, total N excretion per livestock unit is as follows:

   $N_{excretion}$ (g N livestock unit day^{-1}) = 0.50 (50% of total excretion is excreted in bomas) * 69.0 g N day^{-1} = 34.5 g N day^{-1} (range: 18 – 51 g N day^{-1})

f) Each livestock unit occupies 10 m^2 space in a boma and spends 40% of a year in any given boma (=2.5 bomas are used simultaneously). The average time a boma is used is 3.7 years. Both of these assumptions were used for the regional upscaling for SSA and are described in the Supplementary Material).

   Thus, total amount of N excreted in a boma was calculated by:
   $N_{excretion\_total}$ (g N m^{-2}) = 34.5 g N day^{-1} (daily N excretion to a boma) / (10 m^2) * 365/2.5 days yr^{-1} (as 2.5 bomas are used at the same time) * 3.7 yrs (median value of years a boma is used) = 1864 g N m^{-2} (range in dependence of plant N intake: 972 – 2755 g N m^{-2})

g) The cumulative N_2O emission rate from bomas over a 40 years period was calculated as 25 g N_2O-N per m^{-2} boma area (95% confidence interval, CI: 13.3 - 48.1 g N_2O-N m^{-2}). Thus, the emission factor for N_2O over a 40-year period:
   N_2O-EF (40-yr period) = 25 g N_2O-N m^{-2}/1864 g N m^{-2} * 100 = 1.34% (range in dependence of plant N intake: 0.90 – 2.56%)

h) Thus, the N_2O-EF for N cumulated in abandoned bomas equals 1.34% over a 40-year period. This compares directly with the IPCC direct N_2O emission factor of 1% for “Cattle and swine deep bedding” (Table 10.21, IPCC 2019)[Fehler! Textmarke nicht definiert.]. Note, that the IPCC factor reflects N_2O emissions from active bomas, while our estimate reflects N_2O emissions over a

40-year period following abandonment, i.e. both approaches differ with regard to active versus abandoned boma and time scale.

Supplementary Note 2. Correlation analysis between N_2O flux and time since boma abandonment. We used the log_{10}-transformations for the target variable N_2O flux and for the Boma Age to meet the requirement of Gaussian-distributed model residuals with expected values of zero and a common variance (see Supplementary Table 2). Similarly, a constant of 0.5 years was added to the time since abandonment (Boma Age) to enable the log_{10} transformation (Supplementary Table 2).

We built one regression model based on the data from all bomas (each data point was one boma of a certain age) in order to deviate a generalized relationship between boma age and N_2O emissions. From this relationship, we calculated a 'generalized' 40 year average emission rate (as well as its percentiles), which is a emission rate per boma-area (of an 'average' boma, not one specifically for each one of the observed bomas).

Supplementary Note 3. Multiple regression analysis for identifying drivers of boma N_2O fluxes. In a multiple regression analysis, soil temperature was the only variable significantly affecting boma N_2O fluxes ($p<0.05$) while predictors such as soil moisture, soil organic carbon and nitrogen (SOC, SON) content were not significant (Supplementary Table 3). Supplementary Table 4 shows the cross-correlation between individual parameters used in the multiple regression analysis.

Supplementary References

[1] http://www.fao.org/faostat/en/#data/QA, visited in March 2020
[2] http://opendata.rcmrd.org/datasets/africa-isobioclimates
Fehler! Textmarke nicht definiert. Gilbert, M., *et al*. Global distribution data for cattle, buffaloes, horses, sheep, goats, pigs, chickens and ducks in 2010. Sci. Data **5**, 180227 (2018)
[3] https://pir.sa.gov.au/__data/assets/pdf_file/0007/272869/Calculating_dry_matter_intakes.pdf, visited in June 2020
[4] IPCC, Refinement to the 2006 IPCC Guidelines for National Greenhouse Gas Inventories, Chapter 10, Emissions from Livestock and Manure Management. *Intergovernmental Panel on Climate Chane (IPCC), https://www.ipcc-nggip.iges.or.jp/public/2019rf/index.html)*
[5] http://afs.okstate.edu/breeds/cattle/masai/index.html/, visited June 24 2020
[6] Le Roux, X., Mordelet, P., Leaf and canopy CO_2 assimilation in a West African humid savanna during the early growing season. *J. Tropical Ecology* **11**, 529-545 (1995)
