# Supplementary material for: Livestock enclosures in drylands of Sub-Saharan Africa are overlooked hotspots of N2O emissions
Source: Nat Commun. 2020 Sep 15;11:4644. doi: 10.1038/s41467-020-18359-y (PMC7493980; doi:10.1038/s41467-020-18359-y)
Supplement: Supplementary file 2 — Peer Review File [file 41467_2020_18359_MOESM2_ESM.docx]

Peer Review Information

**Journal:** Nature Communications

**Manuscript title:** Livestock enclosures in drylands of Sub-Saharan Africa are overlooked hotspots of N2O emissions

**Corresponding author name(s):** Prof Klaus Butterbach-Bahl

**Editorial notes:**

**Transferred manuscripts**

This manuscript has been previously reviewed at another journal that is not operating a transparent peer review scheme. This document only contains reviewer comments and rebuttal letters for versions considered at *Nature Communications*.

**Reviewer comments & decisions:**

**Reviewer comments, first version:**

Reviewer #1 (Remarks to the Author):

This paper presents N2O flux measurements from a number of abandoned livestock enclosures (and adjacent reference sites) in semi-arid regions of Kenya. Results showed that these enclosures, or bomas, are a significant source of N2O for many years post-abandonment. An upscaling procedure was used to infer that this source may be 10x that of current estimates of manure management emissions from Africa, and 5% of total anthropogenic N2O emissions for the continent. I found this paper to be quite well-written, and the topic to be sufficiently novel to warrant publication in Nature Communications. My comments are generally very minor in nature, though I do take issue with the final claim linking these emissions to the global N2O growth rate and think the authors could do more to place their results in a global context. I recommend acceptance after these are addressed.

Specific comments:

Line 166: “Our results indicate that livestock-related N2O emissions have been severely underestimated using current IPCC methodology.” Assuming IPCC is using the same animal population information used here, I guess this difference comes from lack of specific information about the emission factor associated with this type of manure management system? Can the authors put their results in terms of an emission factor (per amount of N excreted) and compare to those used by IPCC?

Line 170: “Our results show that observed increases in atmospheric N2O concentrations are not only closely linked to the global increase in the use of fertilizers, but also to numbers of livestock in arid and semi-arid regions and the manure accumulation in bomas.” I disagree that the results say this, since this study doesn’t make any claims regarding the change of total boma emissions over time. One could potentially infer the changing flux from the change in animal numbers, but that is not done here. To further put these results in the global context, it would be helpful to compare these results to the literature regarding the contribution of African emissions to the N2O growth rate. Admittedly, this is a difficult thing to determine given the limitations of the global observing system for N2O (e.g., Wells et al., 2015), but the recent paper by Thompson et al. (2019) suggests that African emissions contribute 20% to the recent growth in global N2O emissions. How do these results fit into that estimate? Can the authors compare to published information for growth of fertilizer use and see what flux would be needed from both sources (fertilizer and manure) to contribute 20% of the global flux growth? Doing a little work on this would strengthen the conclusions of the paper and the overall impact of the results.

Line 210-218: I find the discussion of the N2O flux distribution calculation here confusing—it sounds like the authors used the regression model to extrapolate the emission rate over time for each boma investigated, and then calculated a 40 year mean emission rate at each? Is that correct? If not, please consider clarifying.

Figure 3: Can the authors include flux totals as an inset for each map?

Supplementary info: This is a minor comment, but it would help if the figures were arranged in the order in which they are referenced in the main text

Figure S2: This is potentially a nice way to show the evolution of pastoral grazing throughout the day, but I find it difficult to see the animal figures in each panel, and several of the panels look quite similar. Could a few words be added to each panel to denote the main activity happening in each one (i.e. grazing, watering, etc)?

Technical comments:

Line 36: Delete the comma after “systematic”
Line 48: Is “Mio” a standard abbreviation for million? I am not familiar with it.
Line 55: Is the “e.g.” needed here?
Line 87: Delete the quotation marks at the end of the sentence.
Line 99: The range should be included within the parentheses, I think.
Line 116 (Figure 2 caption): Delete the second instance of “Details on the regression are given in Table S1. Also, it looks like the details are actually located in Table S2.
Line 199: “splots” should be “plots”
Line 209: What is AIC?
Line 219: It looks like this paragraph contains some unintended text (“Give the overall approach in descriptive way for clarity. Below, the input variables are listed, but I am looking for…”) I assume the paragraph is actually supposed to start with “Gridded estimates of livestock numbers…”?
Table S3: Some of the units and variable names differ between the equation and table. Can the authors make them consistent?

References:
Wells, K.C., D.B. Millet, N. Bousserez, D.K. Henze, S. Chaliyakunnel, T.J. Griffis, Y. Luan, E.J. Dlugokencky, R.G. Prinn, S. O'Doherty, R.F. Weiss, G.S. Dutton, J.W. Elkins, P.B. Krummel, R. Langenfelds, L.P. Steele, E.A. Kort, S.C. Wofsy, and T. Umezawa (2015), Simulation of atmospheric N2O with GEOS-Chem and its adjoint: evaluation of observational constraints, Geosci. Model Dev., 8, 3179-3198, doi:10.5194/gmd-8-3179-2015.

Thompson, R.L., L. Lassaletta, P.K. Patra, C. Wilson, K.C. Wells, A. Gressent, E.N. Koffi, M.P. Chipperfield, W. Winiwarter, E.A. Davidson, H. Tian, and J.G. Canadell (2019), Acceleration of global N2O emissions seen from two decades of atmospheric inversion, Nat. Clim. Change, 9, 993-998, doi:10.1038/s41558-019-0613-7.


Reviewer #2 (Remarks to the Author):

I have now finished reviewing the manuscript entitled “Livestock enclosures in drylands of Sub-Saharan Africa – overlooked hotspots of N2O emissions”. It is written very well, and the topic is of high importance. Clearly, the bomas areas are hotspots of N2O emissions in that area. The study is rather unique in terms of the sum remote places to be considered as the great sources of emissions. However, some additional information can add to the value of the work. More information about the estimated area of boma sites in compare to whole sub-Saharan Africa would be needed. It would be also helpful if coauthors share their observations of the crust formation on those areas along with organic layer thickness. It would be important to get an estimate of how much the emissions from boma is controlled by the resistance of the surface crust over time. In addition, some mineral N data can be complementary for such a study. I also miss the discussion around the possible solution to distribute that amount of animal excreta to the other areas considering the cost of transport. It would be very useful to make a discussion about the possibility of provided solution as spreading of accumulated manure.


Reviewer #3 (Remarks to the Author):

Review comments on manuscript NCOMMS-20-19079-T

This is a well-written and interesting paper with potential to significantly change the accounting of GHGs for Africa as per current IPCC assumptions and defaults. The statistical approaches and calculations appear sound, but there a few clarification points detailed below that need to be addressed to validate the method used to measure N2O fluxes, and justify some of the choices that were made for calculations:

L30-35. This section is not perfectly clear. Are Bomas of widespread use throughout Africa or a management practice more specific to SSA? In other words, are your estimates (26.3 to 94.2; avr 49.4) for SSA only or estimated for Africa? I believe this is ok to compare the values to Africa's IPCC defaults, even if the values are coming form a specific region, but the reader needs to make sure what is compared to what...

L36. Remove comma after “systematic”.

L86. For sake of clarity, replace "those" with "bomas".

L99. Unclear: it this -0.7 to 15.9? If so, I suggest replacing "-" with "to" before "15.9".

L111. Please clarify "in comparison": I guess the values taken in bomas were corrected for background values in adjacent unperturbed sites? Please make sure this is clear for the reader either in text (e.g., L78-80) or here in this caption.

L127. Awkward phrasing. Suggest replacing “that did not proceed” by “without”.

L191-192. I am not familiar with that particular equipment, and I am wondering what is the impact of this "pressing against" action in the minutes following deployment of these chambers. Have the authors taken into account the possible "transient flush" of N2O escaping the “pressed” organic layer in the minutes following chamber deployment? Such a flush could possibly create an artifact susceptible to substantially increase total flux during the 4-7 min deployment. Please clarify in the text.

L199. Delete extra “s” to “splots”.

L202. What is the rationale for this cut off? Is this because bomas are generally used for less than 2 years? You indicate a central value of 1 y in L254. Please clarify the text and rationale for this cut off here.

L219. Sentence looks awkward. Consider starting with “The overall approach is given..” ?

L259. Phrasing unclear (“elevated N2O fluxes”). Would it be more accurate to say "...with N2O fluxes significantly greater than at adjacent savanna sites..."?

Suppl. Materials, Fig S9 caption, L.114: replace “calculating” with “calculated”.

**Author rebuttal, first version:**

This paper presents N_2_O flux measurements from a number of abandoned livestock enclosures (and adjacent reference sites) in semi-arid regions of Kenya. Results showed that these enclosures, or bomas, are a significant source of N_2_O for many years post-abandonment. An upscaling procedure was used to infer that this source may be 10x that of current estimates of manure management emissions from Africa, and 5% of total anthropogenic N_2_O emissions for the continent. I found this paper to be quite well-written, and the topic to be sufficiently novel to warrant publication in Nature Communications. My comments are generally very minor in nature, though I do take issue with the final claim linking these emissions to the global N_2_O growth rate and think the authors could do more to place their results in a global context. I recommend acceptance after these are addressed.

*Reply: Thank you very much for the positive assessment and the critical points that you raised. Based on these comments, we developed a timeline showing how the magnitude of N_2_O emissions from bomas may have changed over the last decades, with increases in livestock numbers driving these changes. Below please find a point-to-point to reply to the specific comments.*

Specific comments:

Line 166: “Our results indicate that livestock-related N_2_O emissions have been severely underestimated using current IPCC methodology.” Assuming IPCC is using the same animal population information used here, I guess this difference comes from lack of specific information about the emission factor associated with this type of manure management system? Can the authors put their results in terms of an emission factor (per amount of N excreted) and compare to those used by IPCC?

*Reply: We developed a scheme to estimate an EF_N2O_ which was needed to answer this point and included the outcome in the manuscript. The details of the calculation are given in the Supplementary Material. In summary, approximately 1.34% of the manure N accumulated in bomas is finally emitted to the atmosphere in form of N_2_O over 40 years -since abandonment. This number should be considered an additional flux to the estimate of 1% that IPCC uses for “Cattle and swine deep bedding” (Table 10.21, IPCC 2019)**^[1]^, which assumes that all emissions occur while a livestock enclosure (“paddock”) is currently used.*

*Note: we did not subtract background emissions, i.e. N_2_O emissions from undisturbed Savanna sites, as those were marginal, close to the detection limit and thus, assumed to be negligible.*

*Assumptions and calculations:*

a) *Fifty percent of the ingested feed is excreted in bomas as they are used approximately 12 hr day^-1^;*
b) *Cattle dry matter intake is 2% of its body weight, which is an estimate for livestock feeding on dry pasture^[2]^;*
c) *The average weight of a livestock (cattle = 1, sheep and goats = 0.1) unit is 300 kg. This was based on the mean between the IPCC estimate, which assumes mean live weights for dairy and other cattle in Africa to be 260 and 236 kg respectively (IPCC 2019, Chapter 11, Table 10.5), and more refined estimates of Oklahoma State University**^[3]^ who found that Massai livestock were between 360 kg (cows) and 400 kg (bulls).*
d) *Livestock feed comprises mainly tropical grasses, which have a leaf dry matter nitrogen content ranging 0.6 - 1.7% (mean value: 1.15%)^[4]^*.
e) *Based on these numbers we calculated total N intake per cattle as follows:*

*N_intake_ = 300,000 g (body weight) * 0.02 (%DM intake day^-1^) * 0.0115 (%N in dry matter) = 69.0 g N day^-1^ (range 36 – 102 g N day^-1^);*

*We estimate excretion using the assumption that N intake equals N excretion. This is based on the IPCC (2019) data for mature males held in low productivity systems and fed on pastures. Although mature females have a N retention of 8%, we ignored this for the sake of this calculation. Thus, total N excretion per livestock unit is as follows:*

*N_excretion_ (g N livestock unit day^-1^) = 0.50 (50% of total excretion is excreted in bomas) * 69.0 g N day^-1^ = 34.5 g N day^-1^ (range: 18 – 51 g N day^-1^)*

f) *Each livestock unit occupies 10 m^2^ space in a boma and spends 40% of a year in any given boma (=2.5 bomas are used simultaneously). The average time a boma is used is 3.7 years. Both of these assumptions were used for the regional upscaling for SSA and are described in the Supplementary Material).*

*Thus, total amount of N excreted in a boma was calculated by:*

*N_excretion_total_ (g N m^-2^) = 34.5 g N day^-1^ (daily N excretion to a boma) / (10 m^2^) * 365/2.5 days yr^-1^ (as 2.5 bomas are used at the same time) * 3.7 yrs (median value of years a boma is used) = 1864 g N m^-2^ (range in dependence of plant N intake: 972 – 2755 g N m^-2^)*

g) *The cumulative N_2_O emission rate from bomas over a 40 years period was calculated as 25 g N_2_O-N per m^-2^ boma area (95% confidence interval, CI: 13.3 - 48.1 g N_2_O-N m^-2^). Thus, the emission factor for N_2_O over a 40-year period:*

*N_2_O-EF (40-yr period) = 25 g N_2_O-N m^-2^/1864 g N m^-2^ * 100* *=* *1.34% (range in dependence of plant N intake: 0.90 – 2.56%)*

h) *Thus, the N_2_O-EF for N cumulated in abandoned bomas equals 1.34% over a 40-year period. This compares directly with the IPCC direct N_2_O emission factor of 1% for “cattle and swine deep bedding” (Table 10.21, IPCC 2019)^1^. Note, that the IPCC factor reflects N_2_O emissions from active bomas, while our estimate reflects N_2_O emissions over a 40-year period following abandonment, i.e. both approaches differ with regard to active versus abandoned boma and time scale.*

Line 170: “Our results show that observed increases in atmospheric N_2_O concentrations are not only closely linked to the global increase in the use of fertilizers, but also to numbers of livestock in arid and semi-arid regions and the manure accumulation in bomas.” I disagree that the results say this, since this study doesn’t make any claims regarding the change of total boma emissions over time. One could potentially infer the changing flux from the change in animal numbers, but that is not done here. To further put these results in the global context, it would be helpful to compare these results to the literature regarding the contribution of African emissions to the N2O growth rate. Admittedly, this is a difficult thing to determine given the limitations of the global observing system for N2O (e.g., Wells et al., 2015), but the recent paper by Thompson et al. (2019) suggests that African emissions contribute 20% to the recent growth in global N_2_O emissions. How do these
results fit into that estimate? Can the authors compare to published information for growth of fertilizer use and see what flux would be needed from both sources (fertilizer and manure) to contribute 20% of the global flux growth? Doing a little work on this would strengthen the conclusions of the paper and the overall impact of the results.

a) ***Reply:*** *Thank you very much for this critical and constructive comment. We now present a calculation showing how emissions from bomas may have changed over time due to increases in livestock population in semi-arid/ arid regions of SSA in the last six decades and compare with the results presented by Thompson et al. (2019)^6^. This calculation revealed that N_2_O emissions from abandoned bomas likely increased from 23.7 Gg in 1961 (13.9 – 33.2 Gg) to 50.2 Gg in 2015 (29.5 – 70.6 Gg) (lower and upper quartiles). For details see the new Figure 4 in the main manuscript. The calculation is based on the following: The number of total livestock on the African continent from 1961 – 2018 is reported by FAO^[5]^ and graphed in Figure S1;*
b) *The relative distribution of livestock stayed the same over the entire period from 1961 – 2018 as reported in the gridded map by Gilbert et al.^[6]^ for 2010;*
c) *The yearly area of abandoned bomas was calculated based on the livestock numbers in semi-arid/ arid SSA (from a and b) and assigned the model derived from this paper (Figure 2), which shows an exponential decrease in N_2_O emissions over a 40-year period. From this, the annual emissions are highest in the first year following abandonment and decrease to the lowest value after 40 years (and are set to zero in year 41 and following years);*

We used these estimates to assess *whether N_2_O emissions from abandoned bomas are important for explaining recent increases in the source strength of the African continent for atmospheric N_2_O as documented Thompson et al. (2019)**^[7]^ , as per your suggestion.*

*The Thompson et al. (2019) paper states “The increasing trend, however, is probably due to increasing emissions; based on the inversions, emissions increased from 16.3 (15.5–17.1) TgN yr^−1^ for 2000–2005 to 17.9 (17.3–18.5) TgN yr^−1^ for 2010–2015.”. Moreover, the authors state: “The five regions of focus account for ~50% of the global increase between the two time periods,* ***while Africa accounts for ~20%****, Central and South America (excluding Brazil) account for ~10%, Southeast Asia and Oceania account for 8% and 10% was due to changes in ocean emissions”. In other words, they calculated that the global N_2_O emissions* *increased over the 10-year period by 1.6 Tg N yr^-1^, and Africa contributed 20% or 0.32 Tg N yr^-1^ to this increase.*

*Using the calculation above, mean emissions from bomas in semi-arid/ arid SSA increased between* *2000-2005 to 2010-2015 from 38.1 Gg N_2_O yr^-1^ (or 24.2 Gg N yr^-1^) to 47.5 Gg N_2_O yr^-1^ (30.2 Gg N yr^-1^), i.e. by 6 Gg N yr^-1^ or 0.006 Tg N yr^-1^**. The change in boma N_2_O emissions between both periods is equivalent to only ~2% of the 20% change of the source strength of the African continent for atmospheric N_2_O, which is a marginal contribution.*

*Although the contribution of abandoned bomas may be marginal, this research shows that the EF_N2O_ need to be more carefully considered in IPCC recommendations. Specifically, the 1% estimate for “cattle and swine deep bedding” (Table 10.21, IPCC 2019)^1^ is likely a substantial underestimate as we found extremely high N_2_O emissions from bomas abandoned for less than one year (up to 10,266 µg N_2_O-N m^-2^ h^-1^). One would need further research on active bomas, including quantification of N losses via leaching and NH_3_ volatilization, to fully evaluate direct and indirect N_2_O emissions from active bomas.*

*In conclusion, the direct N_2_O emissions from abandoned bomas in semi-arid/ arid environments and the increase due to increased livestock population, does not likely contribute significantly to the increase of the source strength of the African continent to global N_2_O emissions. We did not assess changes in fertilizer use during the study periods, as these data are highly uncertain and have been considered in the gridded N_2_O emission calculations for Africa in EDGAR 5.0^[8]^.*

*We addressed the above discussion in the manuscript by adding the following sentences: “It was recently suggested that Africa contributed 20% to the observed increase in global N_2_O emissions between 2000 – 2005 and 2010 – 2015 (or about 0.32 Tg N yr^-1^ of the total 1.6 Tg N yr^-1^ increase)^6^. Our estimate of the increase in emissions from the abandoned bomas during this period is 0.006 Tg N yr^-1^ (or only 2% of this increase) and therefore cannot explain this difference. However, our measurements from abandoned bomas are biased low, and direct N_2_O emissions from active bomas and indirect N_2_O emissions from leached or volatilized N, which have not been investigated here, may have also contributed to the observed increase in N_2_O emissions from the African continent, and warrants further research”.*

Line 210-218: I find the discussion of the N_2_O flux distribution calculation here confusing—it sounds like the authors used the regression model to extrapolate the emission rate over time for each boma investigated, and then calculated a 40 year mean emission rate at each? Is that correct? If not, please consider clarifying.

***Reply:*** *You are correct, and we clarified this in our revision and added the following information to the Supplementary Material section: “We built one regression model based on the data from all bomas (each data point was one boma of a certain age) in order to deviate a generalized relationship between boma age and N_2_O emissions. From this relationship, we calculated a ‘generalized’ 40 year average emission rate (as well as its percentiles), which is a emission rate per boma-area (of an ‘average’ boma, not one specifically for each one of the observed bomas)*.”

Figure 3: Can the authors include flux totals as an inset for each map?

***Reply:*** *We followed the request of the reviewer and added this information to the maps.*

Supplementary info: This is a minor comment, but it would help if the figures were arranged in the order in which they are referenced in the main text

***Reply:*** *We re-arranged the figures.*

Figure S2: This is potentially a nice way to show the evolution of pastoral grazing throughout the day, but I find it difficult to see the animal figures in each panel, and several of the panels look quite similar. Could a few words be added to each panel to denote the main activity happening in each one (i.e. grazing, watering, etc)?

***Reply:*** *We revised the figure by removing some panels, increasing the pictograms for cattle, and adding text to denote the main livestock activity.*

Technical comments:

Line 36: Delete the comma after “systematic”

***Reply:*** *Deleted*

Line 48: Is “Mio” a standard abbreviation for million? I am not familiar with it.
***Reply:*** *Mio stays for million. Not changed as it is often used.*

Line 55: Is the “e.g.” needed here?

***Reply:*** *Deleted*

Line 87: Delete the quotation marks at the end of the sentence.

***Reply:*** *Deleted*

Line 99: The range should be included within the parentheses, I think.

***Reply:*** *Done*

Line 116 (Figure 2 caption): Delete the second instance of “Details on the regression are given in

***Reply:*** *Deleted*

Table S1. Also, it looks like the details are actually located in Table S2.

***Reply:*** *This is true; it is corrected.*

Line 199: “splots” should be “plots”

***Reply:*** *Corrected*

Line 209: What is AIC?

***Reply:*** *AIC is the Akaike information criterion (AIC), which is an estimator of the prediction error in regression models, and indicates the relative quality of a model for a given data set.*

Line 219: It looks like this paragraph contains some unintended text (“Give the overall approach in descriptive way for clarity. Below, the input variables are listed, but I am looking for…”) I assume the paragraph is actually supposed to start with “Gridded estimates of livestock numbers…”?

***Reply:*** *Corrected. That was a revision error.*

Table S3: Some of the units and variable names differ between the equation and table. Can the authors make them consistent?

***Reply:*** *Thank you for noticing. This is corrected.*

**Reviewer #2**:

I have now finished reviewing the manuscript entitled “Livestock enclosures in drylands of Sub-Saharan Africa – overlooked hotspots of N2O emissions”. It is written very well, and the topic is of high importance. Clearly, the bomas areas are hotspots of N2O emissions in that area. The study is rather unique in terms of the sum remote places to be considered as the great sources of emissions.

***Reply:*** *Thank you very much for the positive comments.*

However, some additional information can add to the value of the work. More information about the estimated area of boma sites in compare to whole sub-Saharan Africa would be needed.

***Reply:*** *For the year 2015 we estimated that the total area of abandoned bomas equals 1792 km^2^. The total area of semi-arid/ arid SSA as defined in this study equals 15.2 Mio km^2^. Thus, the abandoned boma area in 2015 in relation to the total area of arid/ semi/ arid SSA equals* *0.11‰. This information has been added to the “Method Summary” section.*

It would be also helpful if coauthors share their observations of the crust formation on those areas along with organic layer thickness.

***Reply:*** *Crusts consistently form on bomas only if the time since abandonment is <5 years, which is approximately how long it takes for vegetation growth to start (as nutrient concentrations drop below toxic levels), which prevents crust formation. Crusts on bomas abandoned for less than 5 years are usually <5 mm as they are susceptible to rainfall and trampling by wild animals and cattle. The organic layer thickness is indicated in Figure 2, though we only distinguished between organic layer thickness <0.3 m and >0.3 m.* *This threshold value allowed us to distinguish between bomas used consecutively for >2 years compared to those being used for 1 -2 years, because manure accumulates at a rate of about 0.1 to 0.15 m yr^-1^. This information was added to the Methods Summary*

It would be important to get an estimate of how much the emissions from boma is controlled by the resistance of the surface crust over time.

***Reply:*** *The importance of crusts as a control of boma N_2_O emissions was not studied. However, N_2_O emissions from recently abandoned bomas (< 5 years) were on average much higher than from older bomas, which did not have a crust. Thus, the effect of crusts does not seem to be of major importance, specifically as multiple cracks allow for diffusion of gases from deeper parts of the organic layer to the surface. As this remains speculative, we didn’t add this information to the manuscript.*

In addition, some mineral N data can be complementary for such a study.

***Reply:*** *For our sampling we visited rather remote places and mineral N measurements do require immediate extraction. Thus, we only measured total N and C in dried soil samples (which are included in the supplementary data). We recently measured mineral N content in newly-abandoned bomas at the Kapiti site. Thirty days after boma abandonment, NH_4_-N concentrations in the top 5 and 10 cm were 50 -100 µg N g^-1^ dry weight [DW] and 5 - 20 µg N g^-1^ DW respectively. NO_3_-N concentrations in both layers were lower (top 5 cm: 2 - 15 µg N g^-1^ DW; top 10 cm: 0 - 10 µg N g^-1^ DW). In adjacent savanna sites, both NH_4_-N and NO_3_-N concentrations were always well below 5 µg N g^-1^ DW. As this information is rather preliminary, it was not added to the manuscript.*

I also miss the discussion around the possible solution to distribute that amount of animal excreta to the other areas considering the cost of transport. It would be very useful to make a discussion about the possibility of provided solution as spreading of accumulated manure.

***Reply:*** *In our last paragraph, we suggested two options to possibly mitigate N_2_O emissions from bomas:*

a) *To restrict the use of a boma to shorter periods, e.g. one month or less during the wet season. This would reduce the manure and, thus, nutrient accumulation. This will likely reduce legacy effects of the accumulation of manure, not only reducing post-abandonment N_2_O emissions, but also contributing to the fertilization of larger parts of savanna, possibly leading to increased rangeland productivity.*
b) *To recommend that communities spread accumulated manure from longer-used bomas more frequently. This is helpful as some pastoral communities stay in communities for longer time periods and bomas are located in the center of their settlement (see Figure 1c). Given the remoteness of settlements of pastoral communities, there is unlikely to be an economically viable solution for transporting and selling manure.*

*We added the economic perspective as a starting sentence to this paragraph in the main manuscript. Otherwise, the last paragraph does reflect the both points and, thus, we did not add further arguments to the manuscript.*

**Reviewer #3:**

This is a well-written and interesting paper with potential to significantly change the accounting of GHGs for Africa as per current IPCC assumptions and defaults. The statistical approaches and calculations appear sound, but there a few clarification points detailed below that need to be addressed to validate the method used to measure N2O fluxes, and justify some of the choices that were made for calculations.

*Reply: We are grateful for the very constructive comments.*

Line 111, Fig. 2	I guess the values taken in bomas were corrected for background values in adjacent undisturbed sites? Please make sure this is clear for the reader either in text or here in this caption.

***Reply:*** *We did not correct for background fluxes at adjacent undisturbed savanna sites as those were close to zero, with a mean of 5.5 µg N_2_O-N m^-2^ h^-1^ (range: -0.7 – 15.9 µg N_2_O-N m^-2^ h^-1^), compared to fluxes observed at boma sites mean (643 µg N_2_O-N m^-2^ h^-1^, range: 9.3- 10,266 µg N_2_O-N m^-2^ h^-1^). For clarification, we changed the last sentence of the figure caption to: “**Distribution of fluxes for adjacent savanna sites are summarized in a box plot. Given their small magnitude, these were not considered as background in the boma flux calculations.”*

Line 193:	I am not familiar with that particular equipment, but I am wondering what is the impact of this “pressing against” in the minutes following deployment of these chambers. Have the authors taken into account the possible “transient flush” of N_2_O escaping the organic layer immediately after pressing the chamber against the organic layer? Such a flush could possibly create an artifact susceptible to substantially increase total flux during the 4-7 min deployment.

***Reply:*** *This might happen if major pressure differences during chamber deployment occur. However, we used a standard protocol based on Rochette (2011)^[9]^ with vented chambers, in order to avoid overpressure in the chamber headspace (see also Pelster et al., 2017)^[10]^. We added the word “vented” before chambers to clarify this point. In addition, we would see this in the real-time data collection (which is recorded every 5 seconds), and we did not see evidence of this.*

Line 201:	What is the rational for this cutoff? Is this because bomas are generally used for less than 2 years? You indicate a central value of 1 year in L254. Please clarify the text and rational for this cutoff.

*Reply: We edited the sentence as follows to explain the rationale: “A depth of 0.3 m was chosen as a threshold value to distinguish between bomas that were used for 1-2 years and those that were used consecutively for >2 years, as manure accumulates at a rate of 0.1 to 0.15 m yr^-1^.”. Note, there was a typo regarding the central value for the length a boma is used. That should not read “1” but rather “3.7”.*

Line 219:	The overall approach is given…

*Reply: Sentence was deleted as it was a remnant created during a revision.*

Line 259:	Not very clear. Would it be more accurate to say “…with N_2_O fluxes significantly greater than at adjacent savanna sites…”?

***Reply:*** *Changed according to reviewer suggestion. Note, at all sites the boma sites had significantly higher N_2_O fluxes than those from adjacent savanna sites.*

**Reviewer comments, second version:**

REVIEWERS' COMMENTS:

Reviewer #1 (Remarks to the Author):

The authors have addressed all my previous comments in this revision, and I thank them for that. I found just a couple of technical corrections, noted below, after which I recommend publication.

Line 90: "indicated" should be "indicating"

Line 183: The reference number appears to be missing for the Thompson et al. (2019) paper.


Reviewer #2 (Remarks to the Author):

I would like to thank coauthors for the response letter and clarification particularly on the methods and calculations. I thoroughly found this paper to be novel, well-written, and appropriate to consider for publication in Nature Communications.

My comments and the other reviewers' comments were answered with clarity. Therefore,I do not have any further comment.

Dr. Arezoo Taghizadeh-Toosi
Scientist, Department of Agroecology, Aarhus University, Denmark


Reviewer #3 (Remarks to the Author):

I commend the authors for a thorough revision of manuscript. I completely satisfied with the corrections made, and recommend acceptance.

**Author rebuttal, second version:**

**Reviewer #1:**

The authors have addressed all my previous comments in this revision, and I thank them for that. I found just a couple of technical corrections, noted below, after which I recommend publication.

Line 90: "indicated" should be "indicating"

***Reply:*** *This is true; it is corrected.*

Line 183: The reference number appears to be missing for the Thompson et al. (2019) paper.

***Reply:*** *This is true; it is corrected.*

**Reviewer #2:**

I would like to thank coauthors for the response letter and clarification particularly on the methods and calculations. I thoroughly found this paper to be novel, well-written, and appropriate to consider for publication in Nature Communications.

My comments and the other reviewers' comments were answered with clarity. Therefore,I do not have any further comment.

Dr. Arezoo Taghizadeh-Toosi
Scientist, Department of Agroecology, Aarhus University, Denmark

**Reviewer #3:**

I commend the authors for a thorough revision of manuscript. I completely satisfied with the corrections made, and recommend acceptance.

**Reviewer comments, third version:**

**Author rebuttal, third version:**

1. IPCC, Refinement to the 2006 IPCC Guidelines for National Greenhouse Gas Inventories, Chapter 10, Emissions from Livestock and Manure Management*. Intergovernmental Panel on Climate Chane (IPCC),* [*https://www.ipcc-nggip.iges.or.jp/public/2019rf/index.html*](https://www.ipcc-nggip.iges.or.jp/public/2019rf/index.html)*)*
2. <https://pir.sa.gov.au/__data/assets/pdf_file/0007/272869/Calculating_dry_matter_intakes.pdf>, visited in June 2020
3. http://afs.okstate.edu/breeds/cattle/masai/index.html/, visited June 24 2020
4. Le Roux, X., Mordelet, P., Leaf and canopy CO_2_ assimilation in a West African humid savanna during the early growing season. *J. Tropical Ecology* **11**, 529-545 (1995)
5. <http://www.fao.org/faostat/en/#data/QA>, visited in March 2020
6. Gilbert, M., *et al*. Global distribution data for cattle, buffaloes, horses, sheep, goats, pigs, chickens and ducks in 2010. Sci. Data **5**, 180227 (2018
7. Thompson, R.L. et al. Acceleration of global N_2_O emissions seen from two decades of atmospheric inversion. *Nature Climate Change* **9**, 993–998 (2019)
8. Janssens-Maenhout, G., *et al*. EDGAR v4.3.2 Global Atlas of the three major greenhouse gas emissions for the period 1970–2012. *Earth Syst. Sci. Data* **11**, 959–1002 (2019), <https://edgar.jrc.ec.europa.eu/overview.php?v=50_GHG>
9. Rochette, P., Towards a standard non-steady-state chamber methodology for measuring soil N_2_O emissions. *Anim. Feed Sci. Tech*. **166–167**, 141–146 (2011)
10. Pelster, D.E., *et al.* Smallholder African farms in western Kenya have limited greenhouse gas fluxes. *Biogeosciences* **14**, 187-202 (2017)
